# Direct (Hetero)Arylation for the Synthesis of Molecular Materials: Coupling Thieno[3,4-c]pyrrole-4,6-dione with Perylene Diimide to Yield Novel Non-Fullerene Acceptors for Organic Solar Cells

**DOI:** 10.3390/molecules23040931

**Published:** 2018-04-17

**Authors:** Thomas A. Welsh, Audrey Laventure, Gregory C. Welch

**Affiliations:** Department of Chemistry, University of Calgary, 2500 University Drive N.W., Calgary, AB T2N 1N4, Canada; thomas.welsh@ucalgary.ca (T.A.W.); audrey.laventure@ucalgary.ca (A.L.)

**Keywords:** organic dyes, non-fullerene acceptors, direct heteroarylation, bulk heterojunction solar cells, perylene diimide, thieno[3,4-c]pyrrole-4,6-dione

## Abstract

Herein we report on the synthesis of an *N*-annulated perylene diimide (PDI) disubstituted thieno[3,4-c]pyrrole-4,6-dione (TPD) molecular acceptor (PDI–TPD–PDI) by direct heteroarylation (DHA) methods. Three sets of DHA conditions that explore the effects of solvent, temperature, and catalyst were employed to find the optimal conditions for the synthesis of two PDI–TPD–PDI derivatives. We then selected one PDI–TPD–PDI for use as a non-fullerene acceptor in organic solar cell devices with the donor polymer PBDB-T. Active layer bulk-heterojunction blends were modified using several post-deposition treatments, including thermal annealing, solvent vapour annealing, and high boiling solvent additives. It was found that active layers cast from *o*-dichlorobenzene with a 3% *v/v* diphenylether additive yielded films with adequate phase separation, and subsequently gave the best organic solar cell performance, with power conversion efficiencies greater than 3%.

## 1. Introduction

Direct heteroarylation (DHA) is an efficient carbon-carbon (C–C) cross-coupling technique that has significant advantages over traditional methods [1,2,3,4]. While traditional methods require transmetallation reagents to drive aromatic C–C bond formation, DHA can activate C–H and C–Br bonds via a concerted metallation-deprotonation (CMD) mechanism [5,6]. In terms of atom economy, this is tremendously beneficial. Transmetallation reagents for Suzuki [7], Negishi [8], and Stille [9] require boron, zinc, and tin, respectively, which entail extra synthetic steps, by-products, costs, and hazards [5]. In contrast, DHA can be performed under milder conditions with fewer reagents and in fewer steps [10,11]. The substrate scope for DHA is more limited however. Without a transmetallation intermediate, the aromatic C–H bonds of a given substrate must be sufficiently active for coupling to occur. In addition, DHA reactions are often much more sensitive to issues of selectivity and reactivity. As a result, DHA reactions may require further optimization than more traditional reactions [12,13]. 

**Figure 1:**
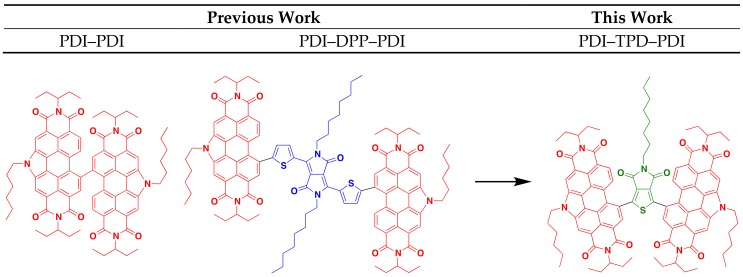
PDI-substituted non-fullerene acceptors previously reported and presented in this work.

There are five primary parameters that can be altered in DHA in order to solve problems associated with activity, selectivity, and reactivity. These are: solvent, temperature, base, acid, and catalyst. In this study, we discuss how these parameters were varied to optimize the syntheses for two molecular materials with an A-A′-A (A = acceptor, an electron deficient π-conjugated unit), where the A′ core is thieno[3,4-c]pyrrole-4,6-dione (TPD) and the A terminal units are *N*-annulated perylene diimides (PDI). Both TPD and PDI have been extensively studied and utilized in materials research, owing to their excellent acceptor properties, high electronic and steric tunability, and ease of functionalization [14,15,16,17]. Many research groups have incorporated TPD in a wide variety of donor-acceptor polymers for bulk heterojunction (BHJ) organic solar cells (OSC). These polymers have demonstrated high power conversion efficiencies (PCE) greater than 8% with fullerene acceptors [18,19,20,21,22,23,24,25,26,27,28]. This is due to TPD’s properties: the monomer’s small steric bulk and low resonance energy provide the polymer with high backbone coplanarity, high electron delocalization, and a low optical band gap [29,30]. The TPD molecule is also easily functionalized at the imide-*N* to tune material solubility and solid-state packing properties. Our research group has used *N*-annulated PDI as terminal units in a number of molecular non-fullerene acceptors (NFA) for BHJ devices (Figure 1) [16,17,31,32,33,34,35]. The *N*-annulated PDI molecule possesses very favorable optoelectronic properties, such as strong visible light absorption, and two easily accessible reduction potentials; these characteristics make PDI an excellent acceptor molecule. 

In our previous research, BHJ OSCs pairing PTB7-Th with a dimer of *N*-hexyl PDI (PDI–PDI, Figure 1) were shown to give PCE of ~5% [33]. This molecule possessed no π-conjugated core and a highly twisted structure. The best OSC devices were fabricated with “as cast” active layers [31,33]. A related structure, PDI–DPP–PDI (DPP = thienyldiketopyrrolopyrrole, Figure 1), bearing an octyl-substituted DPP core group, was used as an acceptor with PTB7-Th, and yielded OSCs with similar PCEs of ~5%. Unique to this system was the need for solvent vapor annealing to organize the active layer and to achieve high performance [16,17]. It was found that the linear octyl chain was key in allowing the molecule to organize into a near ideal nanomorphology [32].

This work focuses on a PDI-TPD-PDI type NFA, which we consider a hybrid of the two NFAs mentioned above. The TPD unit is expected to provide enough steric bulk between the PDI unit to “twist” the structure and prevent significant aggregation, while the central pendant aliphatic group can provide a balance facilitating self-assembly through side-chain interdigitation. While both TPD and PDI have been used in DHA reactions before, they have never been coupled together via DHA. Herein we report on two small molecules synthesized from TPD and PDI via optimized DHA conditions, and explore their optoelectronic properties. TPD units with linear and branched side-chains were explored. Following our previous work, we also investigate how thermal annealing, solvent vapor annealing, and the use of high-boiling solvent additives impact thin film morphologies and subsequent organic solar cell performance [36].

## 2. Results and Discussion

### 2.1. Synthesis and Characterization

**Figure 2:**
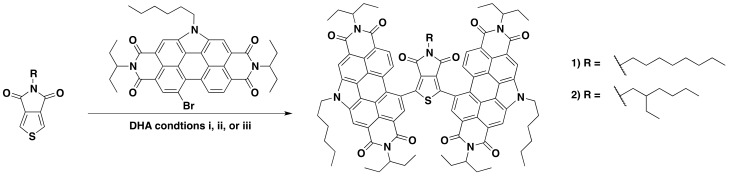
Reaction scheme for the synthesis of compounds **1** and **2**. (i) Toluene, 120 °C, Cs_2_CO_3_, pivalic acid, Pd(Herrmann-Beller), and P(*o*-anisyl)_3_; (ii) DMA, 80 °C, Cs_2_CO_3_, pivalic acid, and Silia*Cat*^®^ DPP-Pd; and iii) toluene, 120 °C, Cs_2_CO_3_, pivalic acid, and Silia*Cat*^®^ DPP-Pd.

In previous studies both TPD and dibrominated-TPD have been functionalized through DHA [14,37]. In our group, we have optimized several DHA reactions with PDI-Br [16,38,39,40]; in this study, we extend this reactivity to the coupling of TPD and PDI–Br together. As mentioned, DHA reaction conditions are very sensitive to the nature of the coupling structures. For this study, we chose to focus on two TPD substrates: 5-octylthieno[3,4-c]pyrrole-4,6,-dione (octTPD) and 5-(2-ethylhexyl)thieno[3,4-c]pyrrole-4,6,-dione (ehTPD) (Figure 2). We began by applying two sets of reaction conditions that have been shown to work well for these substrates. The first set of conditions (i) was developed by the Leclerc group for polymerizing TPD-based substrates via DHA methods [14]. These conditions used toluene as the solvent, a 120 °C reaction temperature, Cs_2_CO_3_ (2–2.5 equiv.) as the base, pivalic acid (30 mol %) as the proton shuttle, and Pd(Herrmann-Beller) (4 mol %) with the ligand P(*o*-anisyl)_3_ (8 mol %) as the catalyst. The second set of conditions (ii) is a standardized DHA method developed by our group for coupling phthalimide- and naphthalimide-based substrates [41], and has since been extended for PDI-Br based DHA couplings [17]. Here, dimethylacetamide (DMA) is used as the solvent, reactions are carried out at 80 °C, K_2_CO_3_ (2–2.5 equiv.) is the base, pivalic acid (30 mol %) is the proton shuttle, and the silica-supported palladium complex Silia*Cat*^®^ DPP-Pd (10 mol % Pd) is used as the catalyst. Use of this catalyst allows for easy metal separation from the reaction media and possible Pd recycling [41,42,43]. In addition to those benefits, the catalyst is also air-stable. For reactions with condition set ii, Cs_2_CO_3_ was used in place of K_2_CO_3_ to allow for a more direct comparison to condition set i.

The two sets of conditions were applied to both TPD units, with two equivalents of PDI-Br, to form (PDI)_2_octTPD (**1**) and (PDI)_2_ehTPD (**2**). Reactions proceeded for 24 h, or until starting materials were no longer visible in the reaction mixture by thin-layer chromatography (TLC). The results are summarized in Table 1. The results show that condition set i worked very well for both TPD substrates, and that products were isolated as solids in similar yields after purification. In contrast, condition set ii did not work at all for either substrate, and only starting materials were obtained after 24 h of reacting time. These results clearly demonstrate the profound effect that the solvent, temperature, and catalyst can have on a DHA reaction. It is interesting to note that, despite involving two equivalents of PDI to one equivalent of TPD, the DHA reaction only proceeded using condition set i, which has been exclusively optimized for TPD by the Leclerc group [37,44]. This indicates that the activation of the C–H bond in the CMD mechanism is more sensitive to reaction conditions than the activation of the C–Br, as would be expected given the relative C–H and C–Br bond energies. Therefore, when considering which DHA conditions to apply to a set of substrates, it is more important to look at the substrate that undergoes C–H, rather than C–Br, activation. 

A third set of conditions were then applied. Set iii combined the solvent and temperature of set i and the Silia*Cat*^®^ DPP-Pd catalyst of set ii. Results from condition set iii are also listed in Table 1. Interestingly, while set iii worked just as well as set i for octTPD, it did not work at all for ehTPD. Only starting materials for this reaction were isolated, similar to reactions with set ii. These results clearly demonstrate the sensitivity of DHA reactions. Here, something as seemingly trivial as an alkyl side chain can constitute the difference between isolating product and starting materials. The reasons for this result are not fully understood, but it is likely that the steric effects of the branched side-chain were preventing interaction between the substrate and the silica-supported catalyst. This will be more fully investigated in future work. Regardless, since both compounds were successfully synthesized using condition set i, a more rigorous approach for investigating the effects of base and acid was not necessary.

### 2.2. Characterization

#### 2.2.1. Optical and Electrochemical Properties

**Figure 3:**
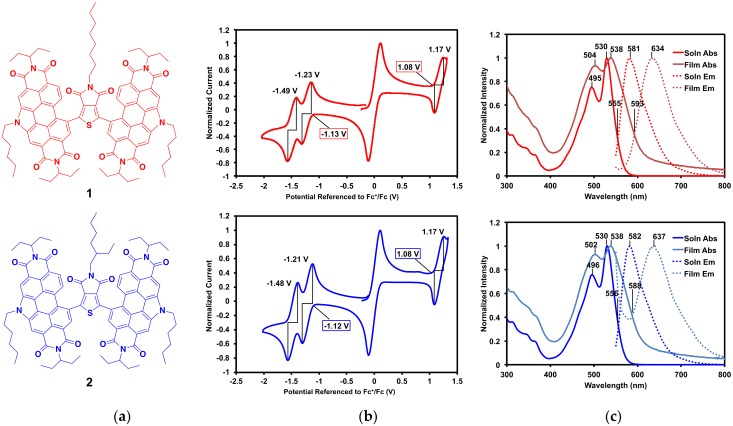
(**a**) Compounds **1** and **2**; (**b**) cyclic voltammograms (with ferrocene internal standard) with onset potentials and E_1/2_ potentials shown; (**c**) solution and thin film absorption and emission profiles with λ_max_ and intercept between absorption and emission profiles, λ_int_, shown. Emission spectra were recorded with excitation at 530 nm.

The optoelectronic properties of compounds **1** and **2** (Figure 3a) were analyzed by cyclic voltammetry and UV-vis absorption and emission spectroscopy. As expected, given that they only differ by the branching of one alkyl chain, **1** and **2** have nearly identical characteristics.

Cyclic voltammograms of **1** and **2** are displayed in Figure 3b. Oxidation and reduction onset and half potentials, ionization potentials (IP), electron affinities (EA), and electrical band gaps (E_elec_) are listed in Table 2. Both compounds exhibit oxidation and reduction waves that are nearly identical. Each compound has one reversible oxidation wave (E_1/2_ = 1.2 V vs. Fc/Fc^+^) and two distinct reduction waves (E_1/2_ = −1.2 and −1.5 V vs. Fc/Fc^+^), which is characteristic of PDI [34]. From the onsets of the oxidation waves and first reduction waves, the IP and EA were calculated, respectively, for each compound. These correspond to an IP of 5.9 eV, EA of 3.7 eV, and an E_elec_ of 2.2 eV, for **1** and **2**. 

The IP and EA of **1** and **2** were then compared to other PDI–π-core–PDI type NFAs that have been synthesized by our group. While EA is determined by the PDI units, the π-core has a profound effect on the IP, and therefore on the overall E_elec_. The most direct comparison can be made with PDI–Th–PDI (Th = thiophene) with an IP of 5.7 eV, EA of 3.5 eV, and E_elec_ of 2.2 eV [34]. Adding an imide group to the thiophene in the core lowers both the IP and EA in **1** and **2**. Compounds with larger π-cores include PDI–DPP–PDI (IP of 5.3 eV, EA of 3.7 eV, E_elec_ of 1.6 eV [16]), PDI–S_2_PO–PDI (S_2_PO = dithienophosphole phenyl oxide, IP of 5.7 eV, EA of 3.6 eV, E_elec_ of 2.1 eV [38]), and PDI–ISI–PDI (ISI = isoindigo, IP of 5.6 eV, EA of 3.6 eV, E_elec_ of 2.0 eV [32]). With a larger core the molecules have lower energy IPs while the EAs are similar. The larger cores allow for a more planar framework and enhanced electron delocalization, while the smaller TPD core leads to a more twisted geometry. As a result, **1** and **2** have overall less π-conjugation and a more electron-poor framework than the larger, more planar compounds. The electronics of **1** and **2** are most similar to PDI–PDI, which lacks a π-core (IP of 6.0 eV, EA of 3.8 eV, E_elec_ of 2.2 eV [31]). This molecule is highly twisted with a PDI–PDI dihedral angle of 96°, similar to the twisting expected in **1** and **2**, though likely not as extreme. Thus, it is clear that while the EA is determined by the PDI units, the IP is heavily influenced by the π-core. These values are tabulated in the supplementary material (SM, Table S2).

The solution and thin film absorption and emission profiles of **1** and **2** are displayed in Figure 3c, and their optical properties including λ_max_, Stokes shifts, and optical band gaps (E_opt_) are listed in Table 2. Complete optical data, including molar absorptivity, is available in the SM. Solution spectra were measured in 2-methyltetrahydrofuran (2-MeTHF) and thin films were spin-cast from 2-MeTHF. This solvent was chosen for its solubility, as well as its potential as a greener processing solvent alternative [45,46]. Both absorption and emission profiles are dominated by the PDI end-caps. In solution, the absorption profiles for both compounds have λ_max_ at 530 nm and the emission profiles have λ_max_ at ~580 nm. This gives large Stokes shifts of 0.21 eV for each compound. In the solid-state, the absorption λ_max_ is ~538 nm and emission λ_max_ is ~635 nm, giving larger Stokes shifts of about 0.35 eV each. Given that Stokes shifts correlate with molecular reorganization energy upon light absorption and emission, a larger Stokes shift in the solid-state, where the molecules are more constrained, is expected. The optical band gaps were calculated from the intercept between absorption and emission profiles, giving 2.2 eV in solution and 2.1 eV in the solid state. These corroborate well with the electrochemical band gaps of 2.2 eV for each compound.

#### 2.2.2. Theoretical Calculations

The PDI–TPD–PDI molecule was also investigated by density functional theory (DFT) calculations. Such calculations, when paired with experimental results, can offer useful insights into structure-property relationships [48,49,50]. An analog for compounds **1** and **2**, one with alkyl chains substituted for methyl units, was investigated in the gas phase by DFT calculations using the B3LYP [51,52,53] level of theory with 6-31G(d,p) [54,55,56,57,58,59] basis set. The optimized geometry was calculated, and from this geometry TD-SCF [60], calculations were performed to generate molecular orbital representations and electrostatic potential maps. We recognize that DFT can overestimate electron delocalization, but the significant twisting within the molecule can mitigate such issues. Full computational data is available in the SM.

**Figure 4:**
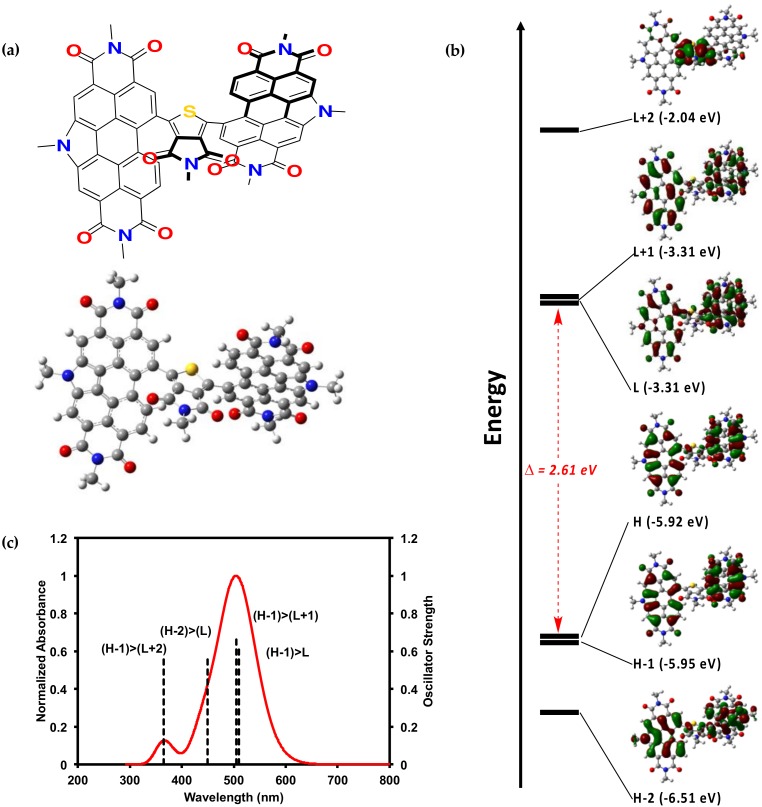
(**a**) Optimized geometry of **1** with methyl groups substituted for alkyl chains; (**b**) calculated electronic energy levels and energy gap for **1**; (**c**) calculated optical absorption profile for **1**. Calculations were done on Gaussian16 [61], input files and results were visualized using GausView05 [62]. All alkyl chains were replaced with a methyl group. The B3LYP [51,52,53] level of theory with 6-31G(d,p) [54,55,56,57,58,59] basis set were used for the calculations. TD-SCF [60] calculations were performed from the optimized geometry. The single point calculation was performed on this structure in order to generate molecular orbitals and electrostatic potential maps.

Figure 4 displays the optimized geometry and calculated molecular orbitals and optical absorption profile. The optimized geometry is extremely twisted with a PDI–TPD dihedral angle of ~50°. The calculated HOMO to LUMO energy gap of 2.5 eV correlates well with the experimental E_elec_ of 2.2 eV. As mentioned above, the electronic properties of **1** and **2** most closely resemble our PDI–PDI, which also possessed a highly twisted geometry with a PDI–PDI dihedral angle of 96°. This lends further credence to the hypothesis that the steric effects of the small TPD core in **1** and **2** influenced the electronics by forcing the molecule to adopt a highly twisted geometry. 

#### 2.2.3. Thin Film Post-Deposition Treatment

**Figure 5:**
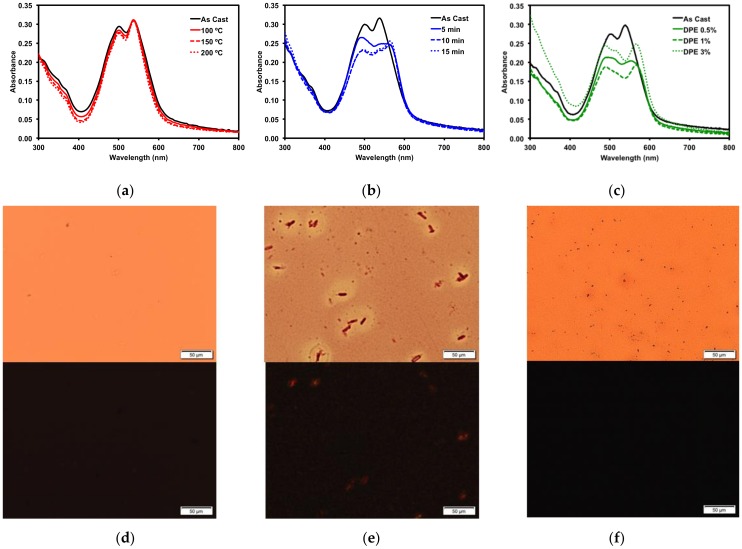
Optical absorption profiles of **1** with (**a**) thermal annealing; (**b**) *o*-DCB solvent vapour annealing; (**c**) DPE solvent additive. POM images of (**d**) 150 °C TA film; (**e**) 15 min *o*-DCB SVA film; and (**f**) 3% *v*/*v* DPE additive film under normal (top) and cross-polarized light (bottom). Images were taken at 20× magnification.

In BHJ solar cells, a certain degree of phase separation in the donor/acceptor blend is necessary to promote electron conductivity and improve performance [31]. It is rare for active layers to possess sufficient phase separation upon casting, so it is often necessary to induce phase separation through post-deposition techniques such as thermal annealing [63], solvent vapour annealing [64], and solvent additives [65]. Post-deposition techniques are applied to evolve the active layer morphology and can serve to increase film density [66]. These additional forces can induce phase separation and possibly improve domain purity through molecular aggregation [36,67]. Thin film morphology evolution from a disordered to a more ordered form can often be observed by large aggregate formation and observed by microscopy imaging and by optical absorption spectroscopy through the emergence of vibrionic fine structure. Therefore, before OSC devices were fabricated, an investigation of various post-deposition techniques was applied to thin films of compound **1**. It has been demonstrated that compounds with linear alkyl chains promote greater organization in active layers and improve device performance [41,68,69,70,71,72,73,74] and therefore only compound **1** with the octyl side chain was investigated. All thin films of **1** were cast from 2-MeTHF. Complete results of these post-deposition techniques are available in the SM and summarized in Figure 5. 

Thermal annealing (TA) provides heat energy to films which can induce reorganization to form a more ordered solid-state morphology [75]. Thin films of **1** were thermally annealed up to 200 °C. No change occurred in the absorption profile (Figure 5a). The films were then analyzed by polarized light optical microscopy (POM), which revealed smooth films with no discernible formation of any new structural features (Figure 5d). The thermal properties were more fully investigated by differential scanning calorimetry (10 °C/min) (SM, Figures S30 and S32) as well as by thermal gravimetric analysis (SM, Figures S31 and S33). Bulk samples of both **1** and **2** showed no indication of melts or crystallizations between 50 °C and 350 °C and the structures were stable up to 400 °C. 

Thin films of **1** were also treated with solvent vapor annealing (SVA) under various solvents (SM, Figure S19). Exposing films to solvent vapors can swell the films and induce reorganization in the morphology to a more ordered state [76]. Of the solvents screened, *o*-dichlorobenzene (*o*-DCB) had the largest impact on the thin films, as determined by optical absorption spectroscopy and microscopy analysis. The UV-vis spectrum (Figure 5b) exhibited a red-shift in the absorption maximum by about 15 nm, indicating that the film morphology had likely undergone a significant reorganization. This was confirmed by POM images, which revealed that SVA from *o*-DCB resulted in the formation of large crystalline features in the film (Figure 5e). Large features such as these usually indicate a large degree of aggregation, which can inhibit OSC device performance.

Three common solvent additives at 1% *v/v* concentrations were then screened with **1** (SM, Figure S22). The addition of high boiling solvents, referred to as volatile additives, to the ink solutions is considered a post-deposition treatment since the volatile additive is presumed to remain after film formation and solvent evaporation, allowing slow evolution of the thin film morphology [27]. Of the additives screened, diphenylether (DPE) had the most dramatic impact on the thin films of **1**. Analysis by optical absorption spectroscopy showed a red-shift in the low energy band and increased vibrionic character (Figure 5c). The emergence of this fine structure in the optical spectra implied the molecule was aggregating, leading to a profound change in the absorption profile. This was further explored by varying the concentration of the DPE additive. Use of 0.5–3% DPE had similar effects (SM, Figures S23 and S24). The POM images revealed that the 3% *v/v* DPE additive resulted in a rougher, but still amorphous, film (Figure 5f). The aggregation in the film induced by the DPE additive was not to the same extent as that observed from the *o*-DCB SVA, which implied that active layers treated with DPE additive would likely exhibit different OSC device performances to those treated with SVA from *o*-DCB. Processing the films with a 5% *v*/*v* concentration of DPE had a profound effect on the film formation (SM, Figure S23). The films appeared foggy, and the absorption was increased significantly across the spectrum, likely due to light scattering. The POM image of the thin-film showed large features evenly distributed across the entire surface (SM, Figure S24).

### 2.3. Organic Solar Cells

#### 2.3.1. Donor/Acceptor Blend Thin Film Post-Deposition Treatment

**Figure 6:**
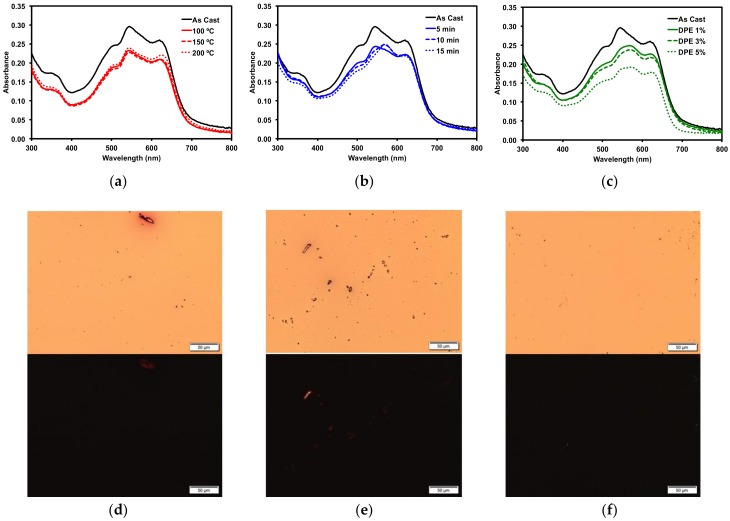
Absorption profiles of 50:50 PBDB-T**/1** blend with (**a**) TA; (**b**) *o*-DCB SVA; (**c**) DPE solvent additive. POM images of (**d**) 150 °C TA film; (**e**) 15 min *o*-DCB SVA film; and (**f**) 3% *v/v* DPE additive film under normal (top) and cross-polarized light (bottom). Images were taken at 20× magnification.

To more accurately determine how the various post-deposition treatments would affect OSC device performance, blends of compound **1** and a common donor polymer were subjected to the same treatment screenings. Bulk-heterojunction blended thin films of **1** and the popular donor polymer PBDB-T (also known as PCE-12) were cast from a 1 wt/*v*% *o*-DCB ink solution with a 50:50 donor/acceptor blend ratio. The thin film optical absorption profiles for the 50:50 blends are shown in Figure 6, along with POM images of selected films. Similar to neat films of **1**, TA had minimal effect on the thin film morphology, as determined by optical spectroscopy and POM, with no changes observed. The SVA treatment using *o*-DCB and processing with the high boiling DPE additive showed changes similar to those observed for neat films of **1** cast from 2-MeTHF. Upon SVA of the film for 10–15 min, a significant change in the optical absorption profile was observed (Figure 6b), with the peak at 570 nm becoming more pronounced. Films processed with 1% and 3% *v/v* DPE additive had a slight decrease in absorption, while processing the films using 5% *v/v* DPE additive gave films with significantly lower absorption, likely due to a thinner film upon spin casting. The POM images showed no significant crystallization of the films, different from the neat films of **1**, which points towards the polymer and **1** forming well-mixed BHJ films.

Based on our findings, four different conditions for active layer processing were selected to test in OSC devices. Films were screened “as cast”, TA at 150 °C, SVA using *o*-DCB for 15 min, and after being processed from solutions with a 3% *v/v* DPE additive. The primary solvent used for ink formulations was *o*-DCB. The “as cast” device would be used as a baseline to gauge the effectiveness of the three post-deposition treatments. Since thermal annealing at the three temperatures had identical effects, the intermediate temperature of 150 °C was chosen. Solvent vapor annealing for 15 min was chosen since it showed the most pronounced change in the optical absorption profile and induced crystallization in neat films of **1** as determined by POM. For the DPE additive, films processed with a 3% *v/v* concentration showed a change in the optical absorption profile, which likely correlated to a morphology change and would give an idea about the role of the solvent additive.

#### 2.3.2. Organic Solar Cell Devices

**Figure 7:**
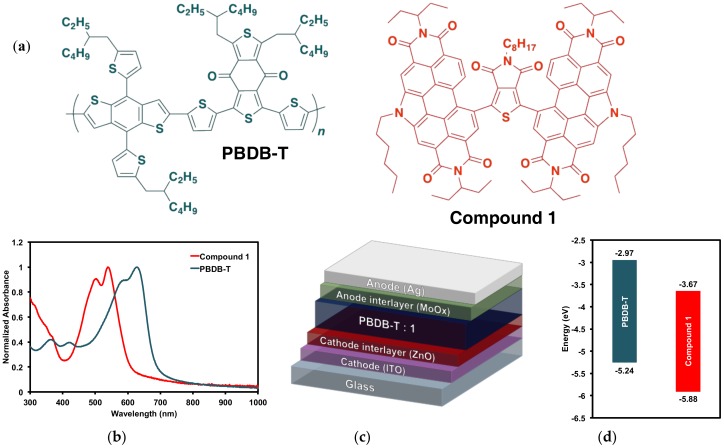
(**a**) PBDB-T donor polymer PBDB-T and (PDI)_2_octTPD (**1**) acceptor; (**b**) thin film absorption profiles; (**c**) device architecture; (**d**) electronic energy levels.

Organic solar cells were fabricated based on our previous work [17,32] following an air-processed and air-tested protocol using an inverted framework: ITO/ZnO/BHJ/MoO_x_/Ag [77] (Figure 7c). The active layers (PBDB-T/**1** 50:50 ratio) were cast at 1500 rpm for 60 seconds from 1 wt/*v*% solutions in *o*-DCB. Active layers were subject to TA at 150 °C for 5 min or SVA with *o*-DCB for 15 min or processed with 3% *v/v* DPE and left to dry for 12 h prior to electrode deposition. Complete results are recorded in the SM, Table S5, and summarized in Table 3 below.

**Figure 8:**
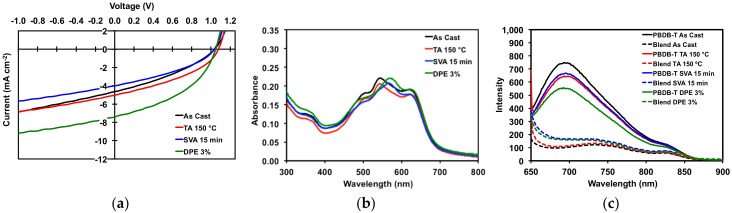
(**a**) JV curves for best devices; (**b**) thin film absorption profiles for 50:50 PBDB-T/**1** blends; (**c**) thin film emission profiles for pure PBDB-T and 50:50 PBDB-T/**1** blends. Plots are for devices as cast, TA at 150 °C for 5 min, SVA from *o*-DCB for 15 min, and with a DPE 3% (*v/v*) additive.

All devices gave high V_OC_ of greater than 1 V. Devices with “as cast” active layers had an average PCE of 1.71%, a modest result. Thermally annealing of the active layers resulted in OSCs with slight increases in J_sc_, FF, and PCE (average of 1.96%). However, the improvements were such that “as cast” and TA devices were more or less equivalent, which corroborated with the optical absorption spectra and POM images for thermally annealed neat and blend films. For OSCs with active layers treated with solvent vapor annealing, performance decreased (average PCE 1.46%) relative to OSCs with “as cast” active layers. Devices with active layers processed with 3% *v/v* DPE additive gave the best overall performance. While V_OC_ remained the same, there was a near doubling of the current with a J_sc_ of 7.02 mA/cm^2^, leading to an increase in average fill factor of 42.85% and an average PCE of 3.14%. Figure 8 displays the JV curves, UV absorption spectra, and photoluminescence spectra for the devices. The photoluminescence spectra (Figure 8c) clearly show that the emission from the neat polymer is quenched in the film, leading to photocurrent generation in the devices. For the devices with active layers treated with SVA and DPE additive, the quenching of emission is less so than with “as cast” and TA devices, as would be expected given the degree of phase separation previously observed in their morphologies. 

**Figure 9:**
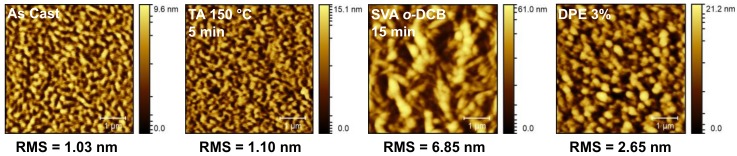
Atomic force microscopy (AFM) images for OSC devices with PBDB-T/**1** (50:50) active layers measured “as cast,” thermally annealed (TA) at 150 °C for 5 min, solvent vapor annealed (SVA) from *o*-DCB for 15 min, and processed with a diphenylether (DPE) 3% *(v/v)* additive.

To probe these results further, atomic force microscopy (AFM) images of the best devices under each condition were acquired. These are presented in Figure 9. The OSC devices with “as cast” and thermally annealed active layers showed relatively smooth films with fine, filamentous domains, which accounts for their similar performances. Active layer films subject to SVA or processed with DPE additive were rougher, especially the SVA film, which showed very large, block-like domains, while the DPE film had smaller, more spherical domains. This indicates that both methods induced phase separation of the two components, with the SVA producing much larger domains. These results correlated well with the POM images of the thin films of compound **1** (Figure 5e,f), where the SVA induced the formation of large crystalline features in the film, while DPE at 3% concentration produced a rougher film with smaller and more regular features. As mentioned above, phase separation is often necessary to improve device performance but too much separation can reduce performance. This is exactly what has been observed here, where the small and regularly dispersed domains formed by the DPE additive increased the device performance while the large and infrequent domains formed by SVA hindered performance.

## 3. Materials and Methods

### 3.1. Materials

Silia*Cat*^®^ DPP-Pd was purchased from SiliCycle (Québec, QB, Canada). 5-octylthieno[3,4-c]pyrrole-4,6,-dione, 5-(2-ethylhexyl)thieno[3,4-c]pyrrole-4,6,-dione, and PBDB-T polymer were purchased from Brilliant Matters. *N*-annulated perylene diimide was synthesized according to our previously reported literature procedure [31]. All remaining reagents were purchased from Sigma-Aldrich (St. Louis, MO, USA). All solvents and materials purchased were used without further purification. 

### 3.2. Characterization

^1^H and ^13^C{^1^H}-NMR spectroscopy spectra were recorded on a Bruker Avance-500 MHz spectrometer (Billerica, MA, USA) at 300 K. Spectra were measured in CDCl_3_ solution and shifts are referenced to tetramethylsilane (CH_3_)_4_Si. All electrochemical measurements were performed using a Model 1200B Series Handheld Potentiostat by CH Instruments Inc (Austin, TX, USA). equipped with Ag wire, Pt wire and glassy carbon electrode, as the pseudo reference, counter electrode and working electrode respectively. All absorption measurements were recorded using Agilent Technologies Cary 60 UV-Vis spectrometer (Santa Clara, CA, USA) at room temperature. All emission measurements were recorded using an Agilent Technologies Cary Eclipse fluorescence spectrophotometer (Santa Clara, CA, USA) at room temperature. Solution experiments were run in 2-methyltetrahydrofuran and neat films were prepared by spin-coating ~0.1 mL from a 1 wt/*v*% solution (2-MeTHF or blend solvents) onto clean Corning glass micro slides (Corning, NY, USA). Polarized optical microscopy experiments (Shinjuku, Tokyo, Japan) were performed using an Olympus Bx53 microscope.

### 3.3. Synthesis and Device Fabrication

#### 3.3.1. Synthesis of (PDI)_2_octTPD (**1**)

In a 5 mL pressure vial were combined 5-octylthieno[3,4-c]pyrrole-4,6,-dione (25 mg, 0.094 mmol, 1.0 eq.), 11-bromo-5-hexyl-2,8-bis(1-ethylpropyl)perylene diimide (133 mg, 0.19 mmol, 2.0 eq.), cesium carbonate (75 mg, 0.23 mmol, 2.5 eq.), and pivalic acid (40 mol %). Herrmann-Beller catalyst (25 mol % Pd) and P(*o*-anisyl)_3_ (19 mg, 0.054 mmol, 0.58 eq.) were added under N_2_. The vial was then sealed with a Teflon^®^ cap (Bristol, RI, USA) and 1.5 mL of degassed, anhydrous toluene were syringed into the vial under N_2_. The mixture was heated to 120 °C in a LabArmor^®^ (Cornelius, OR, USA) bead bath for 18 h. The mixture was then taken up in MeOH and stirred at room temperature for 1 h. This slurry was then filtered through celite and rinsed with MeOH to remove excess toluene. The dark red residue left on the celite was extracted with DCM. The DCM was removed by evaporation, leaving the crude product. The product was purified by precipitation from a stirred CHCl_3_ solution with a slow drip of EtOH (5:1 EtOH/CHCl_3_) and isolating by vacuum filtration (red solid, 68 mg, 0.045 mmol, 49%). ^1^H-NMR (500 MHz, CDCl_3_): δ 9.17 (s, 3H), 9.11 (s, 3H), 8.81 (br s, 2H), 8.69 (br s, 2H), 5.24 (m, 4H), 4.99 (t, 4H, ^3^*J* = 7.1 Hz), 3.41 (br s, 2H), 2.37 (m, 8H), 2.26 (pent, 4H, ^3^*J* = 7.4 Hz), 2.01 (m, 8H), 1.49 (m, 4H), 1.41 (m, 4H), 1.32 (sex, 4H, ^3^*J* = 7.1 Hz), 1.10 (br m, 12H), 0.98 (m, 24H), 0.89 (t, 6H, ^3^*J* = 7.3 Hz), 0.73 (t, 3H, ^3^*J* = 6.9 Hz). ^13^C-NMR (125 MHz, CDCl_3_, ppm): δ 161.8, 145.1, 135.3, 135.2, 133.6, 132.8, 132.1, 127.8, 126.9, 125.8, 125.3, 123.3, 123.1, 120.0, 119.8, 58.2, 58.0, 47.2, 38.9, 31.8, 31.5, 29.1, 29.0, 28.2, 27.1, 26.8, 25.4, 22.7, 22.6, 14.1, 11.6. MS (MALDI-TOF): *m/z* 1514.6767, 1538.6910 [M + Na]. calcd. 1515.70.

#### 3.3.2. Synthesis of (PDI)_2_ehTPD (**2**)

In a 5 mL pressure vial were combined 5-(2-ethylhexyl)thieno[3,4-c]pyrrole-4,6,-dione (25 mg, 0.094 mmol, 1.0 eq.), 11-bromo-5-hexyl-2,8-bis(1-ethylpropyl)perylene diimide (134 mg, 0.19 mmol, 2.0 eq.), cesium carbonate (73 mg, 0.22 mmol, 2.4 eq.), and pivalic acid (40 mol %). Herrmann-Beller catalyst (29 mol % Pd) and P(*o*-anisyl)_3_ (28 mg, 0.080 mmol, 0.85 eq.) were added under N_2_. The vial was then sealed with a Teflon^®^ cap and 1.5 mL of degassed, anhydrous toluene were syringed into the vial under N_2_. The mixture was heated at 120 °C in a LabArmor^®^ bead bath for 22 h. The mixture was then taken up in MeOH and stirred at room temperature for 1 h. This slurry was then filtered through celite and rinsed with MeOH to remove excess toluene. The dark red residue left on the celite was extracted with DCM. The DCM was removed by evaporation leaving the crude product. The product was purified by precipitation from a stirred CHCl_3_ solution with a slow drip of MeOH (5:1 MeOH/CHCl_3_) and isolating by vacuum filtration (red solid, 79 mg, 0.052 mmol, 56%). ^1^H-NMR (500 MHz, CDCl_3_): δ 9.17 (s, 3H), 9.11 (s, 3H), 8.78 (br s, 2H), 8.69 (br s, 2H), 5.24 (m, 4H), 4.99 (t, 4H, ^3^*J* = 7.2 Hz), 3.27 (br s, 2H), 2.37 (m, 8H), 2.26 (pent, 4H, ^3^*J* = 7.4 Hz), 2.01 (m, 8H), 1.50 (m, 4H), 1.41 (m, 4H), 1.32 (sex, 4H, ^3^*J* = 7.2 Hz), 1.09 (br m, 9H), 0.98 (m, 24H), 0.89 (t, 6H, ^3^*J* = 7.3 Hz), 0.70 (m, 3H), 0.62 (br m, 3H). ^13^C-NMR (125 MHz, CDCl_3_, ppm): δ 162.1, 145.1, 135.4, 135.2, 133.5, 133.0, 132.2, 127.8, 126.8, 125.8, 125.4, 123.4, 123.1, 120.0, 119.9, 58.2, 58.0, 47.3, 42.6, 38.0, 31.8, 31.6, 30.4, 29.9, 27.1, 25.4, 24.0, 23.8, 23.0, 22.7, 14.2, 14.1, 11.7. MS (MALDI-TOF): *m/z* 1514.6952, 1538.6978 [M + Na]. calcd. 1515.70.

#### 3.3.3. Device Fabrication

Devices were fabricated using ITO-coated glass substrates cleaned by sequentially ultra-sonicating detergent and de-ionized water, acetone, and isopropanol, followed by exposure to UV/ozone for 30 min. ZnO was subsequently deposited as a sol-gel precursor solution in air, following the method of Sun et al. [77]. The room temperature solution was filtered and spin-cast at a speed of 4000 rpm, and then annealed at 200 °C in air for 15 min. Active layer solutions of PBDB-T (Brilliant Matters, Québec, QB, Canada), PCE12, M_w_ = 154 kg/mol and M_n_ = 76 kg/mol, batch no BM3-009-6), and **1** were prepared in air with a total concentration of 10 mg/mL in *o*-DCB, with or without a 3% *(v/v)* DPE additive. Solutions were stirred overnight at room temperature and heated for 4 h at 80 °C. Active layer materials were combined in a 1:1 weight ratio and cast at room temperature in air at a speed of 1500 rpm for 60 sec. Thermal annealing was done for 5 min at 150 °C when indicated. Solvent vapor annealing from *o*-DCB was done for 15 min. All substrates upon casting active layers were kept in an N_2_ atmosphere glovebox overnight before evaporating MoO_3_ and Ag. The evaporation of 10 nm of MoO_3_ followed by 100 nm of Ag were thermally deposited under vacuum (3 × 10^−6^ Torr). The active areas of resulting devices were 0.09 cm^2^. For each result 4 substrates (2 devices per substrate) were prepared.

## 4. Conclusions

We have reported on the syntheses of two electron deficient molecules comprised of a TPD core with PDI terminal units using direct heteroarylation methods. The compounds with the general structure PDI–TPD–PDI only differed by topology of the alkyl chain on the central TPD unit. The efficiency of coupling reactions was found to be primarily dependent on catalyst and solvent selection. The PDI–TPD–PDI molecule, compound **1**, bearing an octyl chain on the TPD unit was investigated as a non-fullerene acceptor in bulk-heterojunction solar cells. The thin film morphology of this material was found to be robust against thermal annealing but sensitive to solvent vapor annealing and the use of processing additives. Exposure of films of **1** to *o*-DCB solvent vapor induced crystallization of the thin films and produced large aggregates. Processing films of **1** using the diphenyl ether solvent additive had a subtle effect, giving small aggregates dispersed evenly across the entire film. Preliminary organic solar cells fabricated using the PDI–TPD–PDI acceptor and the donor polymer PBDB-T could give power conversion efficiencies above 3% with films processed using the diphenyl ether solvent additive. Notably, the photovoltaic cells had high open-circuit voltages >1 V. This work has identified the TPD unit to be a useful building block for the construction of non-fullerene acceptors using direct heteroarylation methods. Next steps will focus on organic solar cell optimization using this acceptor in combination with a variety of donor polymers, with an emphasis on processing films using solvent additive, to achieve higher efficiencies.

## Figures and Tables

**Table 1:** Reaction results for conditions i, ii, and iii with octTPD and ehTPD. (i) Toluene, 120 °C, Cs_2_CO_3_, pivalic acid, and Pd(Herrmann-Beller, and P(*o*-anisyl)_3_; (ii) DMA, 80 °C, Cs_2_CO_3_, pivalic acid, and Silia*Cat*^®^ DPP-Pd; and (iii) toluene, 120 °C, Cs_2_CO_3_, pivalic acid, and Silia*Cat*^®^ DPP-Pd.

TPD R Group	Conditions	Yield (%) ^1^
octyl	i	49
octyl	ii	0
octyl	iii	46
ethylhexyl	i	56
ethylhexyl	ii	0
ethylhexyl	iii	0

^1^ Yields were ~50% due to product lost during the purification process. See the Materials and Methods section.

**Table 2:** Summary of experimental optoelectronic properties of compounds **1** and **2**.

Property	Compound 1	Compound 2
E_Ox_ Onset (V)	1.08	1.08
E_1/2_ Ox (V)	1.17	1.17
E_Red_ Onset (V)	−1.13	−1.12
E_1/2_ Red (V)	−1.23, −1.49	−1.21, −1.48
IP (eV) ^1^	5.88	5.88
EA (eV) ^1^	3.67	3.68
E_elec_ (eV)	2.21	2.20
Solution λ_abs_ (nm)	530	530
Solution λ_em_ (nm)	581	582
Solution E_opt_ (eV) ^2^	2.24	2.23
Solution Stokes Shift (eV) ^3^	0.21	0.21
Thin Film λ_abs_ (nm)	538	538
Thin Film λ_em_ (nm)	634	637
Thin Film E_opt_ (eV) ^2^	2.09	2.11
Thin Film Stokes Shift (eV) ^3^	0.35	0.36

^1^ Energies were calculated by (Onset V + 4.8) where Fc HOMO = 4.8 eV [47]. ^2^ Optical band gaps were calculated from the intercept of absorption and emission profiles where (E_λint_ = h × c/λ_int_; h = Planck’s Constant, c = speed of light). ^3^ Stokes Shifts were calculated by (E_λabs_ – E_λems_) where (E_λmax_ = h × c/λ_max_).

**Table 3:** Optimized organic solar cell data for 50:50 PBDB-T/**1** blends ^1^.

Parameters	V_OC_ (V) Avg. (Best)	J_sc_ (mA/cm^2^) Avg. (Best)	FF (%) Avg. (Best)	PCE (%) Avg. (Best)
As Cast	1.06 (1.07)	4.66 (4.84)	34.66 (35.15)	1.71 (1.81)
TA 150 °C 5 min	1.07 (1.07)	4.91 (5.04)	37.14 (38.59)	1.96 (2.09)
SVA *o*-DCB 15 min	1.03 (1.03)	3.92 (4.00)	36.33 (36.52)	1.46 (1.50)
DPE 3%	1.04 (1.05)	7.02 (7.40)	42.85 (42.37)	3.14 (3.28)

^1^ Device architecture: ITO/ZnO/BHJ/MoO_x_/Ag. Active layers were spin cast at 1500 rpm from 10 mg/mL solutions in *o*-DCB.

## Supplementary Materials

Supplementary materials are available online.
