# Supplementary material for: Direct (Hetero)Arylation for the Synthesis of Molecular Materials: Coupling Thieno[3,4-c]pyrrole-4,6-dione with Perylene Diimide to Yield Novel Non-Fullerene Acceptors for Organic Solar Cells
Source: Molecules. 2018 Apr 17;23(4):931. doi: 10.3390/molecules23040931 (PMC6017723; doi:10.3390/molecules23040931)
Supplement: Supplementary file 1 [file molecules-23-00931-s001.pdf]

Direct (hetero)arylation for the synthesis of molecular materials: Coupling thieno[3,4-c]pyrrole-4,6-dione with perylene diimide to yield novel non-fullerene acceptors for organic solar cells

Thomas A. Welsh, Audrey Laventure, Gregory C. Welch*

Department of Chemistry, University of Calgary
2500 University Drive NW Calgary, AB, Canada T2N 1N4

*Corresponding Author
Email: gregory.welch@ucalgary.ca
Phone Number: 1-403-210-7603

SUPPORTING INFORMATION

Table of Contents

**Materials and Methods**	**S2**
**Solution NMR Spectra**	**S4**
**Mass Spectrometry (MALDI-TOF)**	**S5**
**Elemental Analysis**	**S7**
**Electrochemical Characterization**	**S9**
**Optical Absorption Characterization**	**S12**
**Thin Film Treatments - Thermal Annealing**	**S15**
**Thin Film Treatments - Solvent Vapour Annealing**	**S16**
**Thin Film Treatments - Volatile Solvent Additives**	**S17**
**BHJ Blends**	**S18**
**Thermal Characterization**	**S20**
**Theoretical Modelling**	**S22**
**Organic solar cells**	**S24**
**References**	**S25**

1. Materials and Methods

High-resolution Mass Spectrometry (HRMS): High-resolution MALDI mass spectrometry measurements were performed courtesy of Jian Jun (Johnson) Li in the Chemical Instrumentation Facility at the University of Calgary. A Bruker Autoflex III Smartbeam MALDI-TOF (Na:YAG laser, 355nm), setting in positive reflective mode, was used to acquire spectra. Operation settings were all typical, e.g. laser offset 62-69; laser frequency 200Hz; and number of shots 300. The target used was Bruker MTP 384 ground steel plate target. Sample solution (~ 1 µg/mL in dichloromethane) was mixed with matrix trans-2-[3-(4-tert-Butylphenyl)-2-methyl-2-propenylidene]malononitrile (DCTB) solution (~ 5mg/mL in methanol). Pipetted 1µl solution above to target spot and dried in the fume hood.

Density Functional Theory (DFT): Calculations were carried out using Gaussian16 [1], input files and results were visualized using GausView05 [2]. All alkyl chains were replaced with a methyl group. The B3LYP level of theory with 6-31G(d,p) basis set were used for the calculations. TD-SCF [12] calculations were performed from the optimized geometries. Single point calculations were performed on optimized structures in order to generate molecular orbitals.

Power Conversion Efficiency (PCE): The current density-voltage (J-V) curves were measured in air by a Keithley 2420 source measure unit. The photocurrent was measured under AM 1.5 illumination at 100mW/cm^2 under a Solar Simulator (Newport 92251A-1000). The standard silicon solar cell (Newport 91150V) was used to calibrate light intensity.

Atomic Force Microscopy (AFM): AFM measurements were performed by using a TT2- AFM (AFM Workshop) in tapping mode and WSxM software with a 0.01-0.025 Ohm/cm Sb (n) doped Si probe with a reflective back side aluminum coating. Samples for AFM measurements were the same ones that were used to collect the respective device parameters and EQE profiles.

2. Solution NMR Spectra

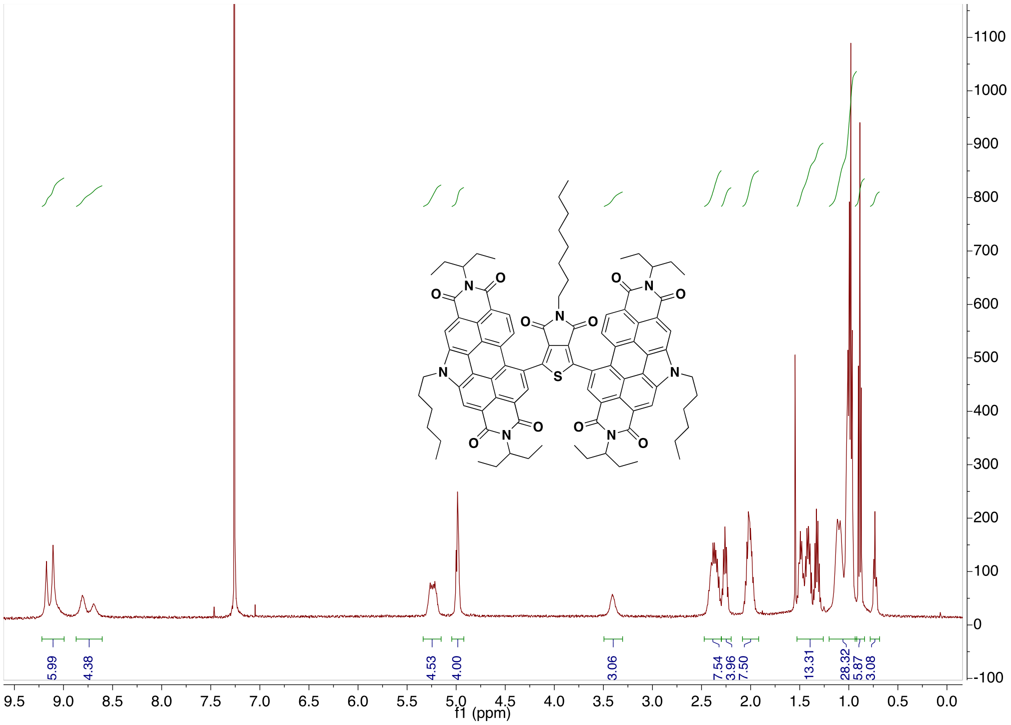

Figure S1: ^{1}H NMR spectrum of **1** in $CDCl_3$.

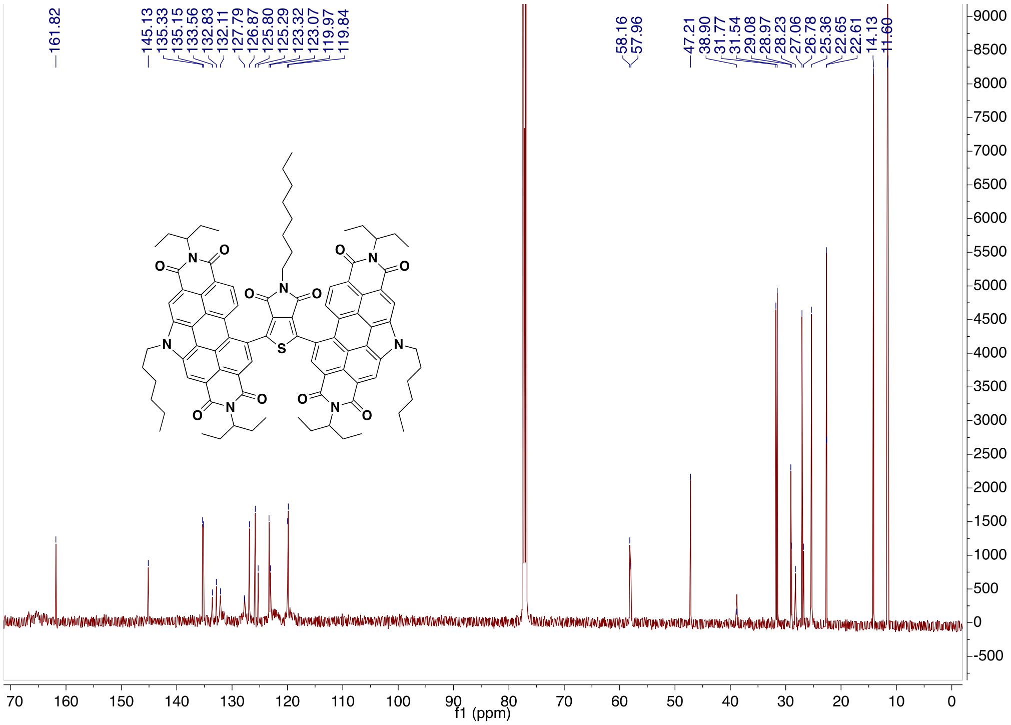

Figure S2: ^{13}C NMR spectrum of **1** in $CDCl_3$.

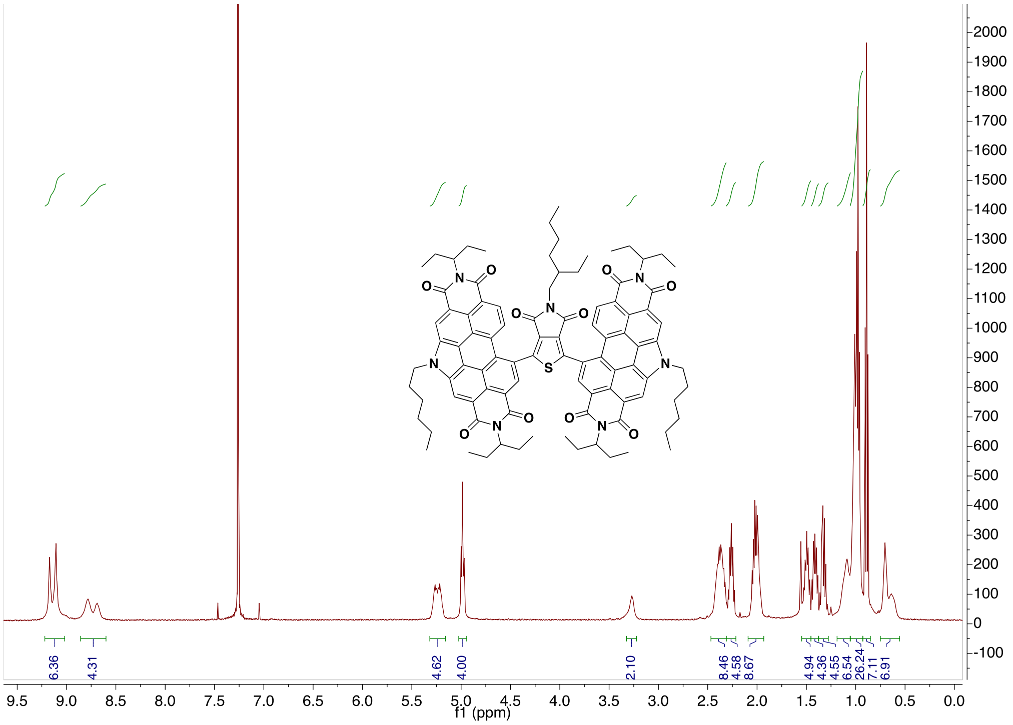

Figure S3: ^{1}H NMR spectrum of **2** in $CDCl_3$.

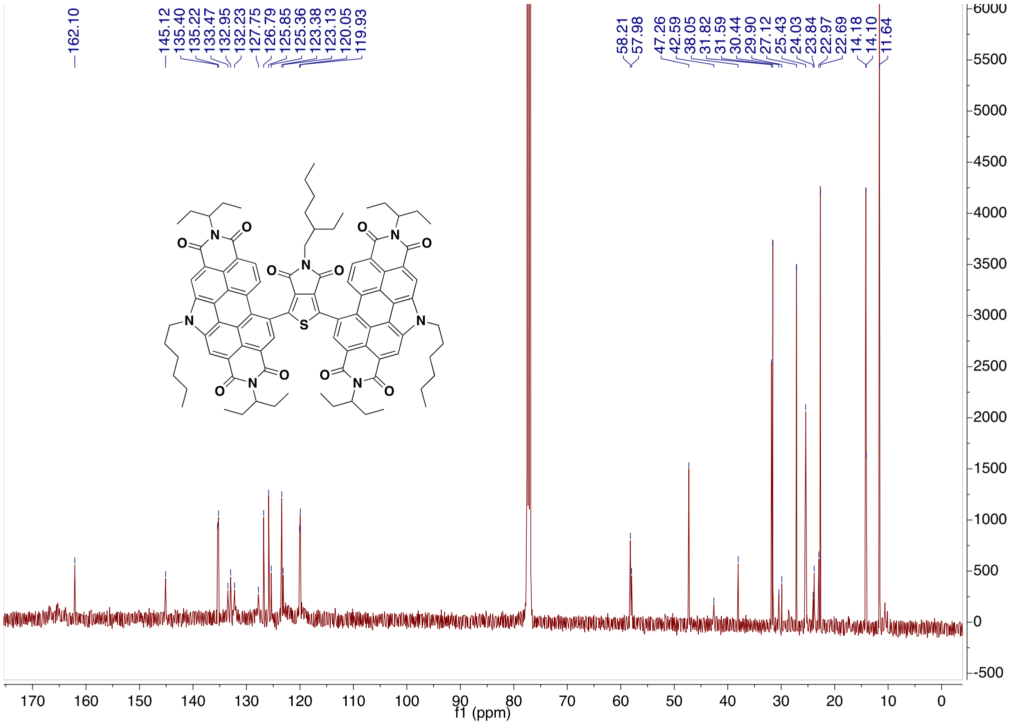

Figure S4: ^{13}C NMR spectrum of **2** in $CDCl_3$.

3. Mass Spectra (MALDI-TOF)

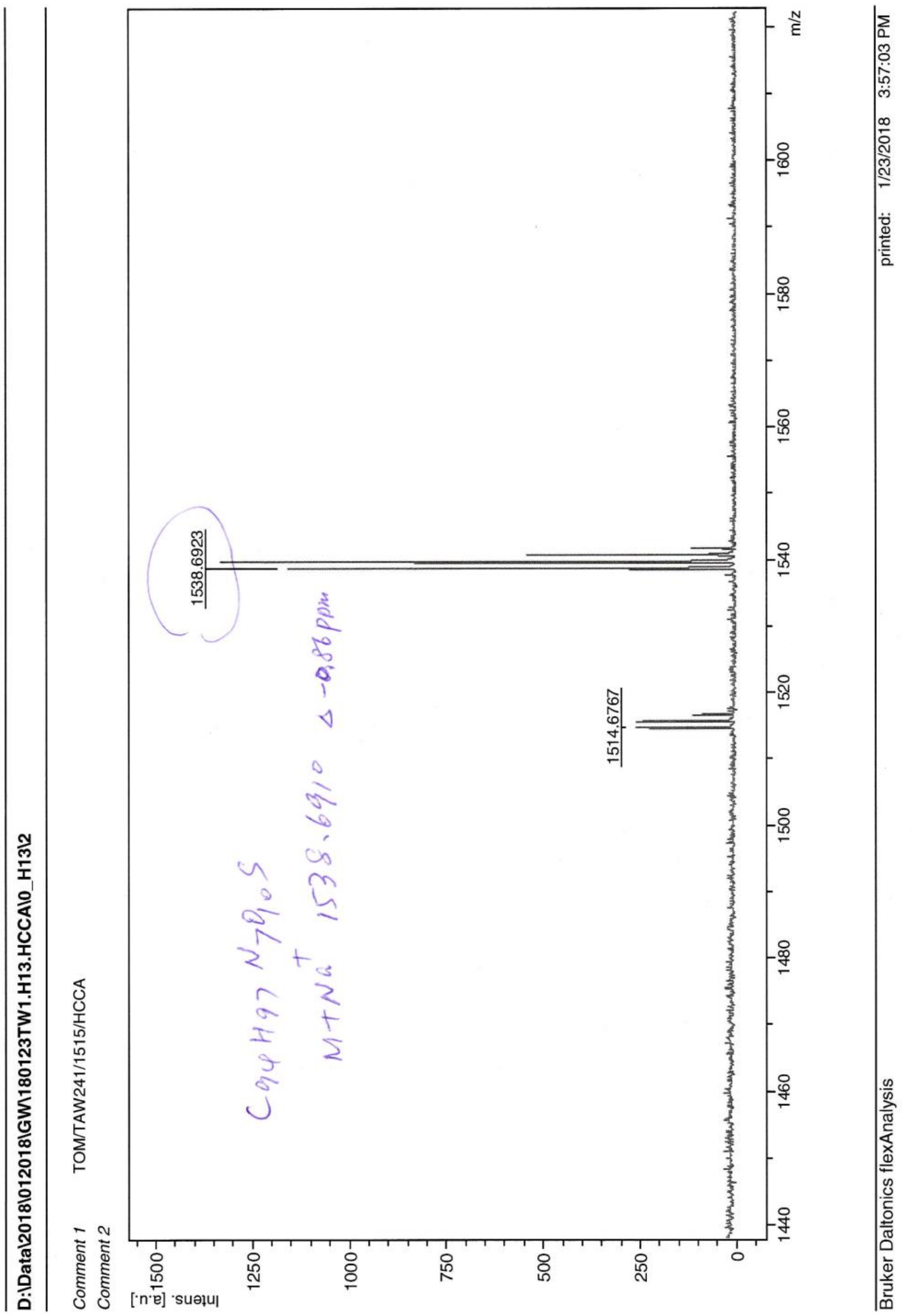

Figure S5: MALDI-TOF of **1**.

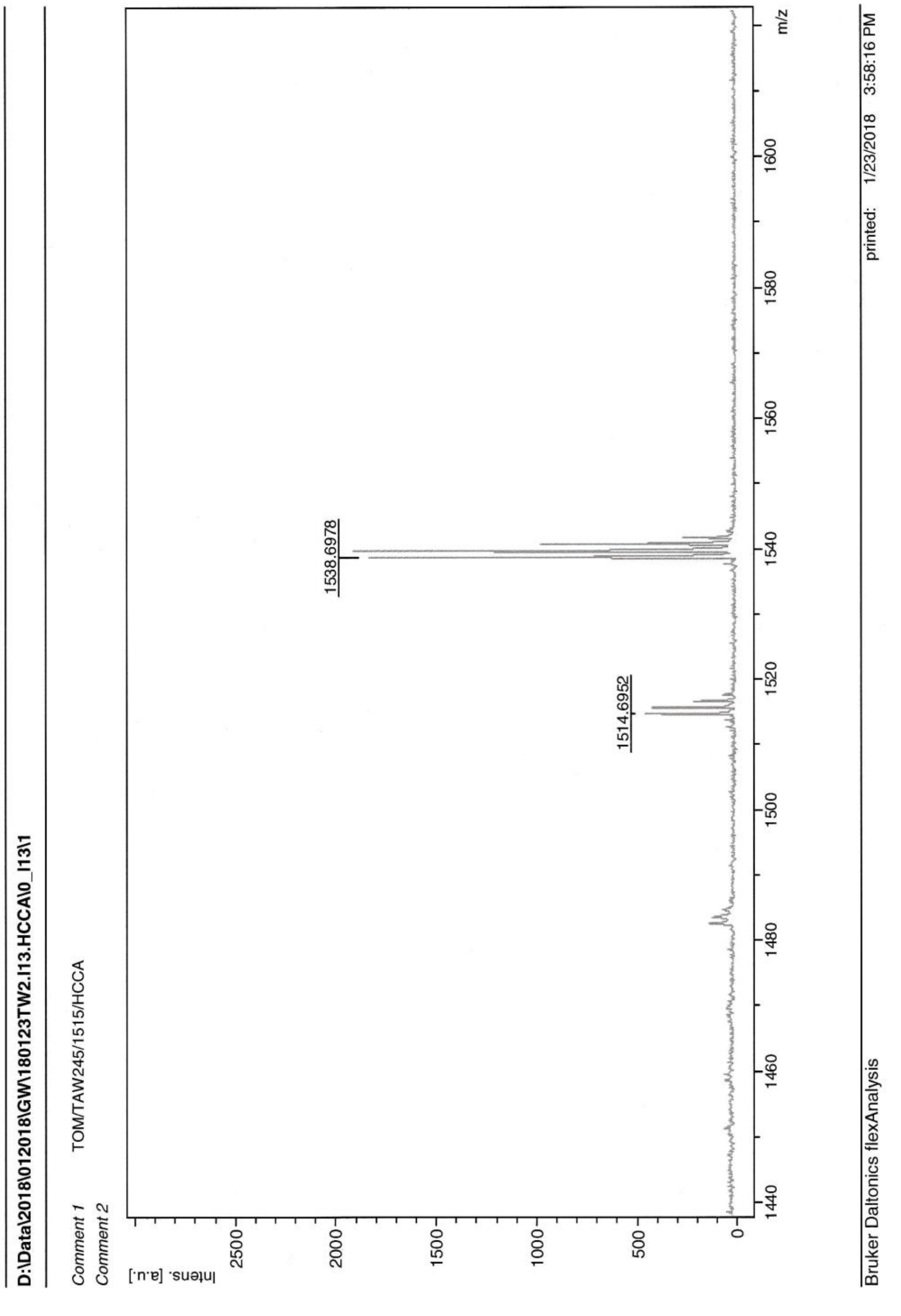

Figure S6: MALDI-TOF of **2**.

4. Elemental Analysis

University of Calgary
Department of Chemistry EA Date: 1/24/2018

Name:	TOM	Group:	GW
Sample:	TAW241-1	Weight (mg):	1.183
%C (Actual):	74.00	%C (Theorectical):	74.43
%H (Actual):	6.33	%H (Theoretical):	6.45
%N (Actual):	6.07	%N (Theoretical):	6.46

University of Calgary
Department of Chemistry EA Date: 1/24/2018

Name:	TOM	Group:	GW
Sample:	TAW241-2	Weight (mg):	1.079
%C (Actual):	73.65	%C (Theorectical):	74.43
%H (Actual):	6.33	%H (Theoretical):	6.45
%N (Actual):	6.08	%N (Theoretical):	6.46

Figure S7: Elemental analysis results of **1**. Note: %C results are lower than theoretical due to incomplete combustion of perylene diimide units.

University of Calgary

Department of Chemistry EA Date: 1/24/2018

Name:	TOM	Group:	GW
Sample:	TAW245-1	Weight (mg):	1.43
%C (Actual):	73.70	%C (Theorectical):	74.43
%H (Actual):	6.18	%H (Theoretical):	6.45
%N (Actual):	6.09	%N (Theoretical):	6.46

University of Calgary

Department of Chemistry EA Date: 1/24/2018

Name:	TOM	Group:	GW
Sample:	TAW245-2	Weight (mg):	1.682
%C (Actual):	73.35	%C (Theorectical):	74.43
%H (Actual):	6.24	%H (Theoretical):	6.45
%N (Actual):	6.06	%N (Theoretical):	6.46

Figure S8: Elemental analysis results of **2**. Note: %C results are lower than theoretical due to incomplete combustion of perylene diimide units.

5. Electrochemical Characterization

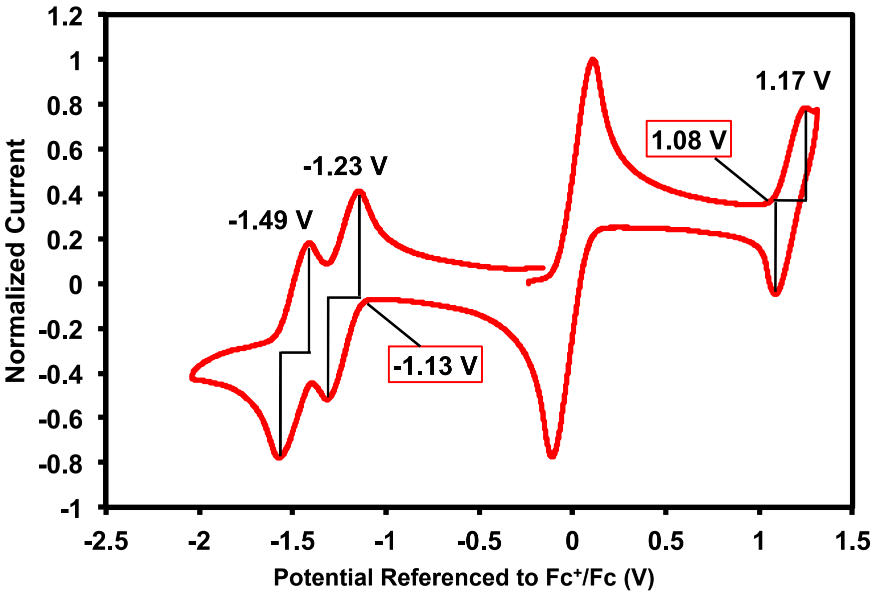

Figure S9: Cyclic voltammogram of **1**.

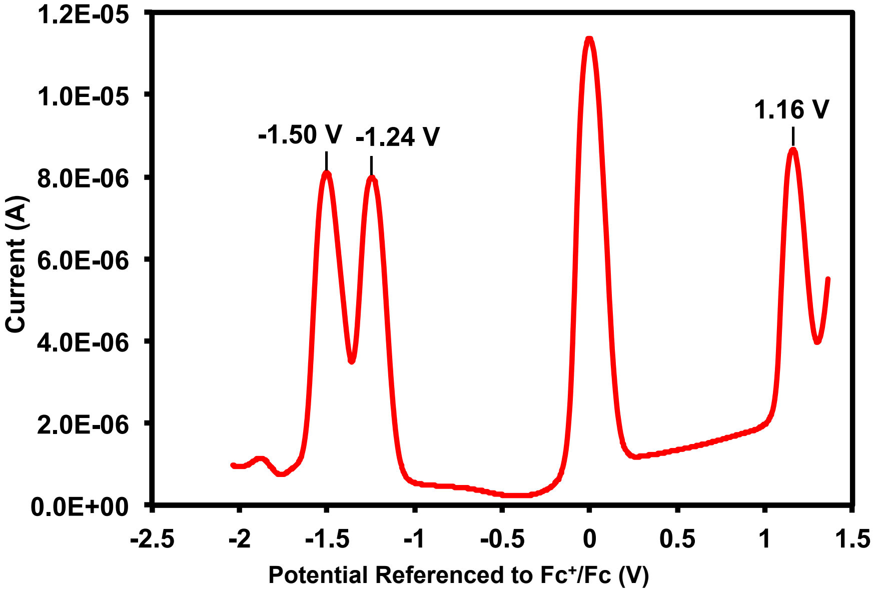

Figure S10: Differential pulse voltammogram of **1**.

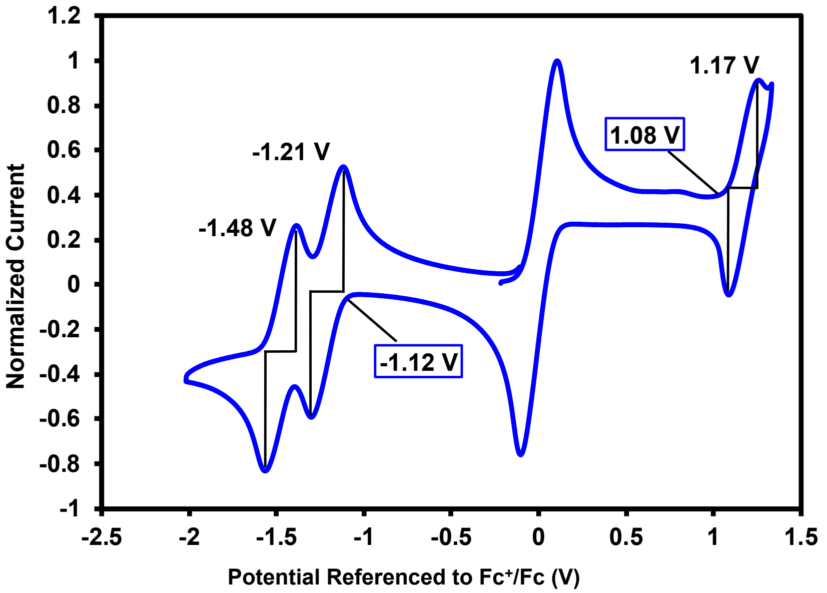

Figure S11: Cyclic voltammogram of **2**.

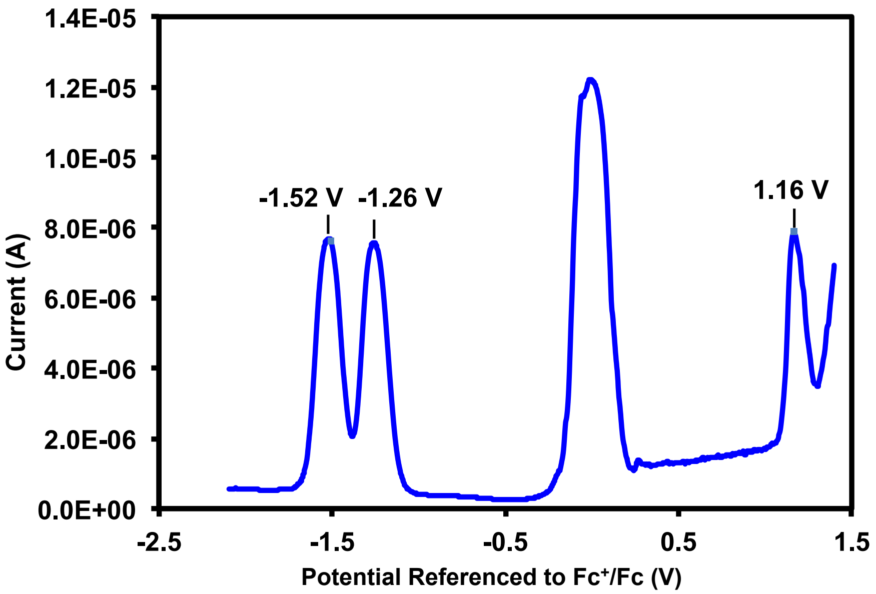

Figure S12: Differential pulse voltammogram of **2**.

Table S1: Summary of electronic properties for **1** and **2**.

	**1**	**2**
E_{Ox} Onset (V)	1.08	1.08
$E_{1/2}$ Ox (V)	1.17	1.17
E_{Red} Onset (V)	-1.13	-1.12
$E_{1/2}$ Red (V)	-1.23, -1.49	-1.21, -1.48
IP (eV)[a]	-5.88	-5.88
EA (eV)[a]	-3.67	-3.68
E_g (eV)	2.21	2.20

[a]Energy values were calculated by (Onset V + 4.8) where 4.8 eV is HOMO of ferrocene [13].

Table S2: Comparison of electrochemical properties of PDI–π-core–PDI type molecules.

***π*-core**	**IP (eV)**	**EA (eV)**	**E_{elec} (eV)**
TPD	5.9	3.7	2.2
Th	5.7	3.5	2.2
DPP	5.3	3.7	1.6
S_2PO	5.7	3.6	2.1
ISI	5.6	3.6	2.0
None	6.0	3.8	2.2

6. Optical Absorption - Solution

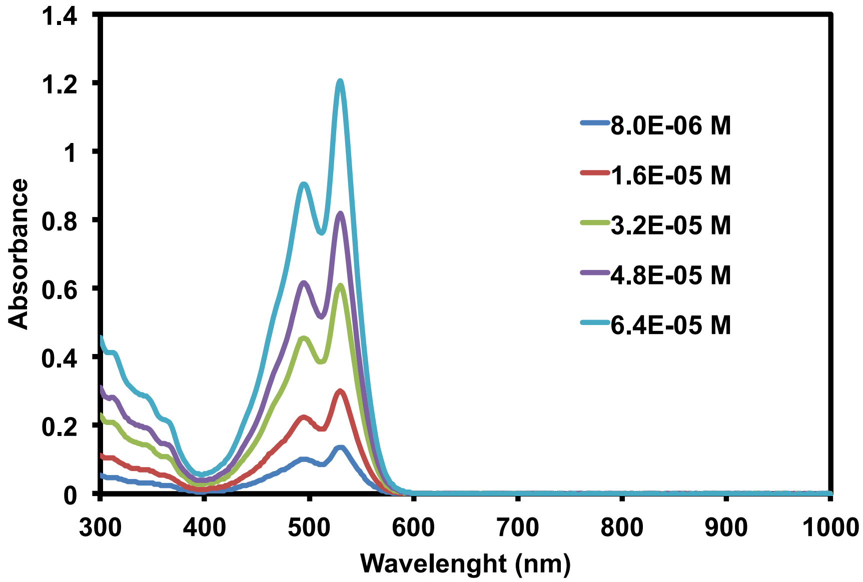

Figure S13: Solution absorption spectra for **1** in 2Me-THF at varying concentrations.

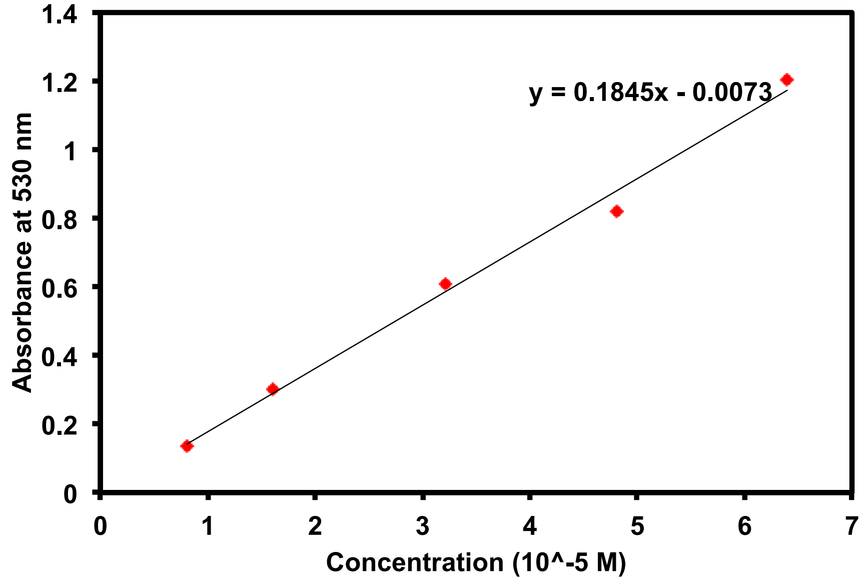

Figure S14: Absorbance versus concentration profile for **1**.

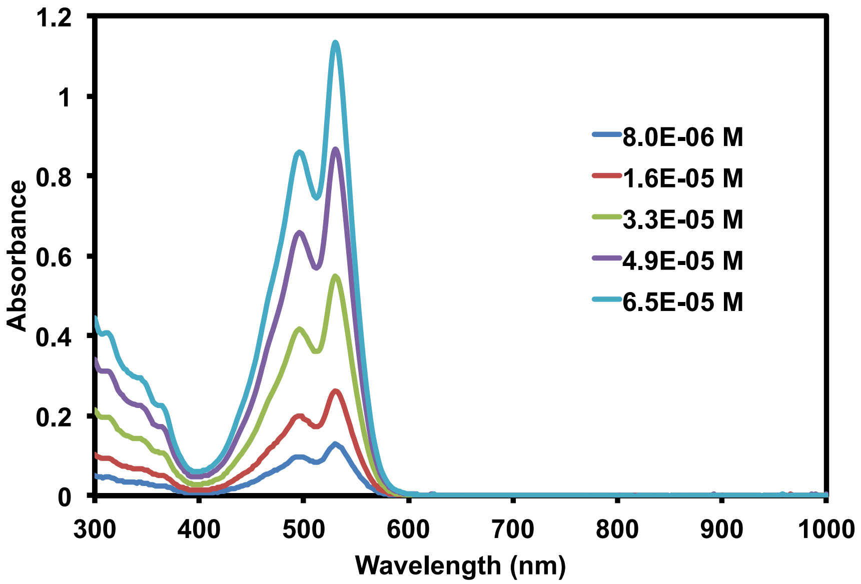

Figure S15: Solution absorption spectra for **2** in 2Me-THF at varying concentrations.

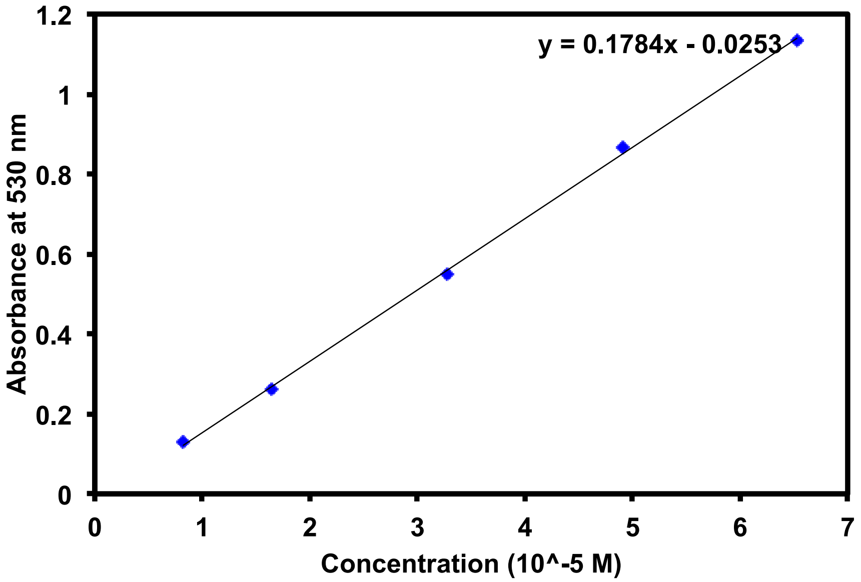

Figure S16: Absorbance versus concentration profile for **2**.

Table S3: Summary of optical properties for **1** and **2**.

	**1**	**2**
Solution Absorbance Max (nm)	530	530
Solution Emission Max (nm)	581	582
Solution Optical E_g (eV)[a]	2.24	2.23
Solution Stokes Shift (eV)[b]	0.21	0.21
Molar Absorptivity (L mol^{-1} cm^{-1})	92274	89212
Thin film Absorbance Max (nm)	538	538
Thin film Emission Max (nm)	634	637
Thin film Optical E_g (eV)[a]	2.09	2.11
Thin film Stokes Shift (eV)[b]	0.35	0.36
Excitation Wavelength (nm)	530	530

[a]Optical band gaps were calculated from the wavelength intercept of absorption and emission profiles where ($E_{\lambda int} = h*c/\lambda_{int}$; h = Planck's Constant, c = speed of light). [b]Stokes Shifts were calculated by ($E_{\lambda abs} – E_{\lambda ems}$) where ($E_{\lambda max} = h*c/\lambda_{max}$).

7. Thin Film Treatments – Thermal Annealing

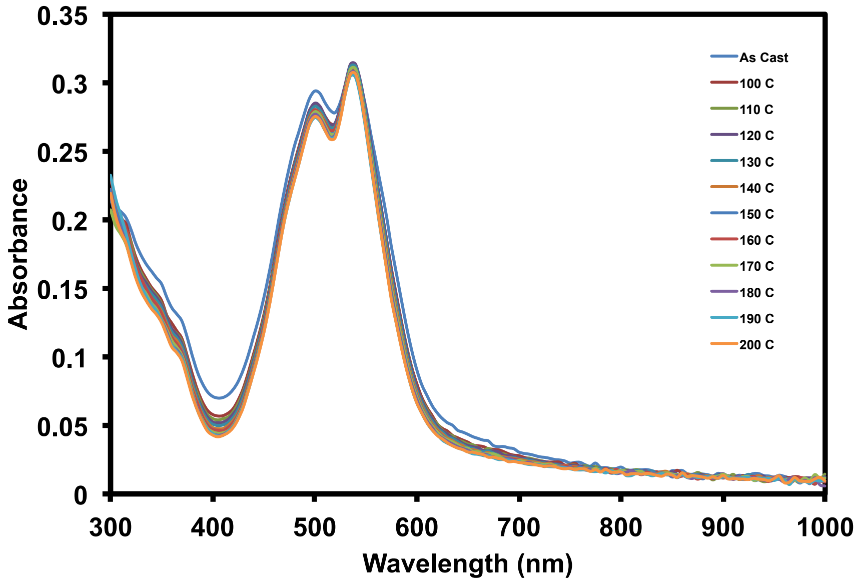

Figure S17: Optical absorption spectra of thin-films of **1** measured "as-cast" and after thermal annealing for five minutes at each temperature. Films where spin-cast from 10 mg/mL 2-MeTHF solutions at 1500 rpm for 30 s.

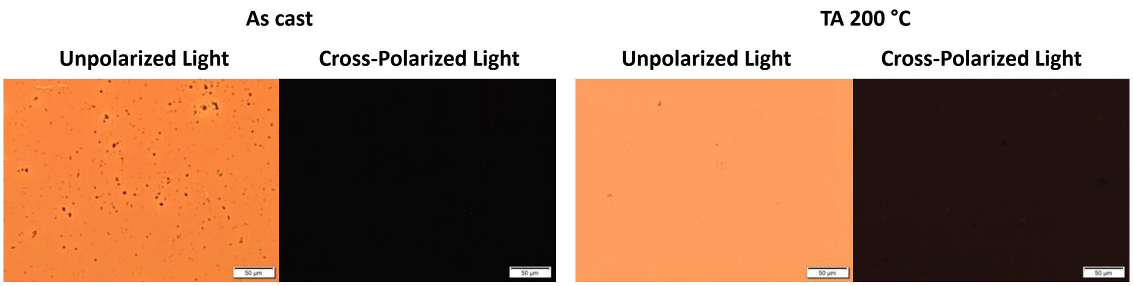

Figure S18: Polarized optical microscopy (POM) images of thin-films of **1** measured "as-cast" and after being thermally annealed up to 200 °C. Images taken under normal and cross-polarized light. Images were taken at 20× magnification. Thermal annealing caused no visible changes in films up to 200 °C.

7. Thin Film Treatments – Solvent Vapour Annealing

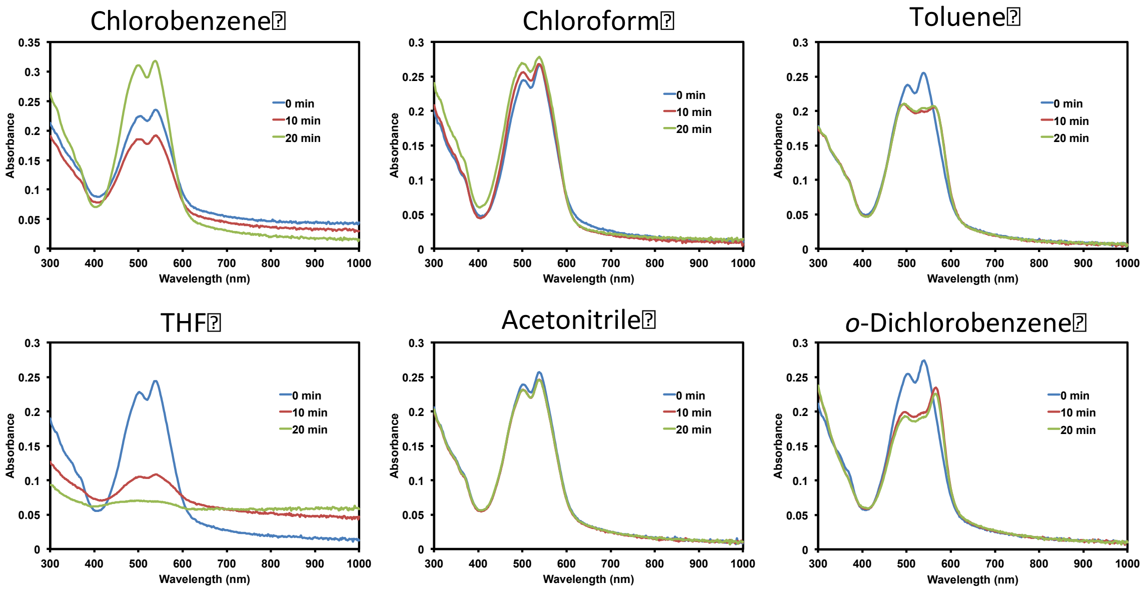

Figure S19: Optical absorption spectra of film of **1** measured "as-cast" and after being solvent vapour annealed from various solvents. Films were exposed to the various solvents for 10 min and 20 min.

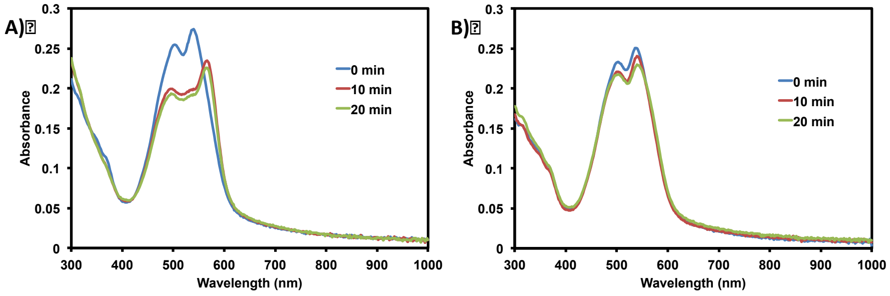

Figure S20: Optical absorption spectra of films measured "as-cast" and after being solvent vapour annealed using *o*-dichlorobenzene (*o*-DCB). A) compound **1** and B) compound **2**.

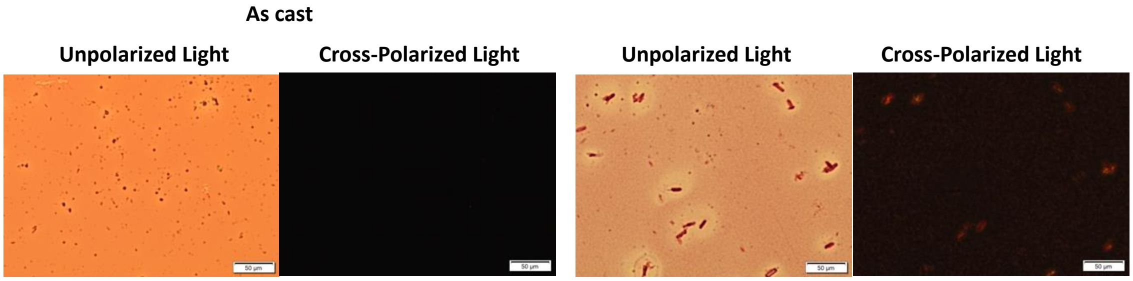

Figure S21: POM images of films of **1** measured "as-cast" and after being solvent vapour annealed with *o*-DCB for 15 min. Images taken under normal and cross-polarized light. Images were taken at 20× magnification.

7. Thin Film Treatments – Volatile Solvent Additives

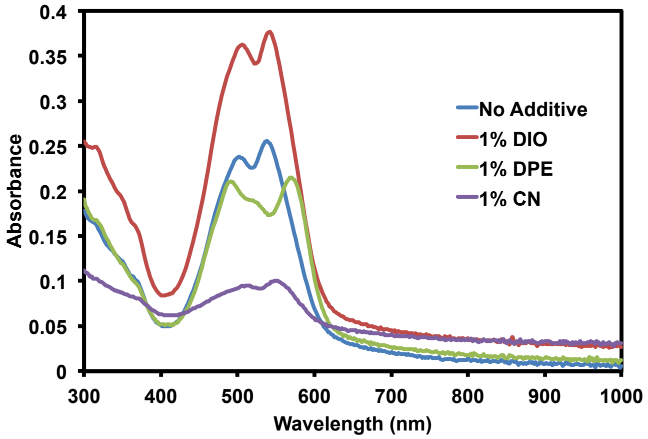

Figure S22: Optical absorption spectra of films of **1** spin-cast from 10 mg/mL 2-MeTHF solutions with 1,8-diiodooctane (DIO), diphenylether (DPE), or 1-chloronaphthalene (CN) additives at 1% v/v concentration.

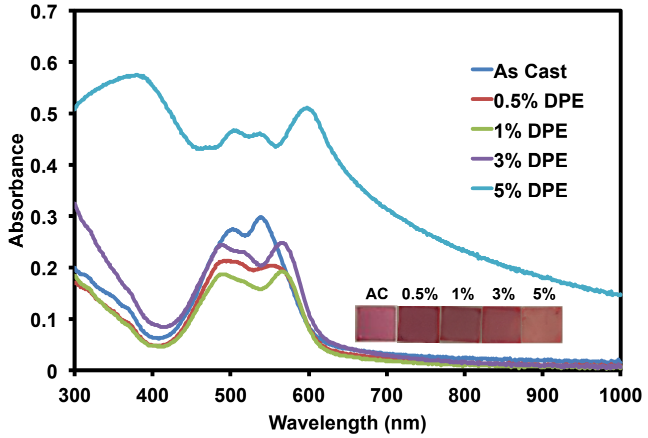

Figure S23: Optical absorption spectra of films of **1** spin-cast from 10mg/mL 2-MeTHF solutions with various concentrations (v/v) of DPE additive. Photos of the thin films are also shown.

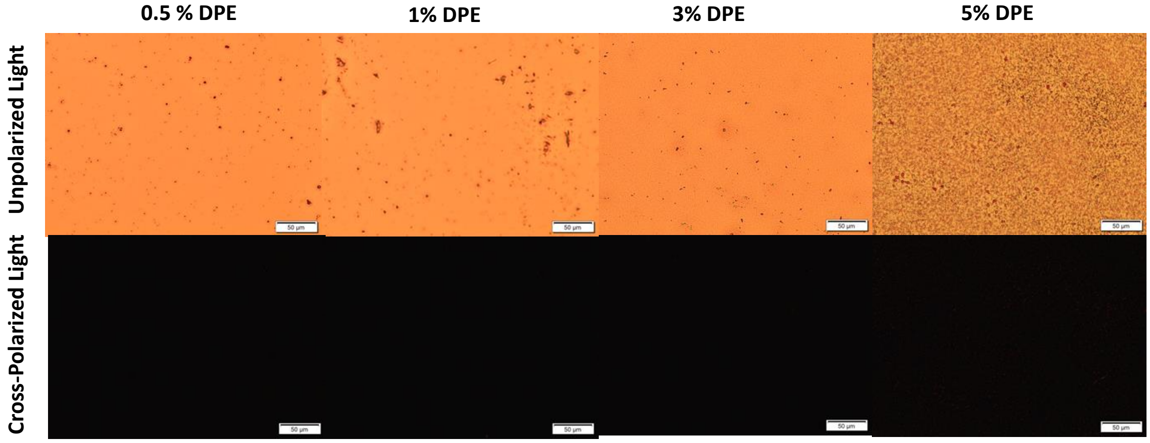

Figure S24: POM images of thin-films of **1** processed with DPE solvent additive. Images taken under normal and cross-polarized light. Images were taken at 20× magnification.

8. BHJ Blends (PBDB-T:1)

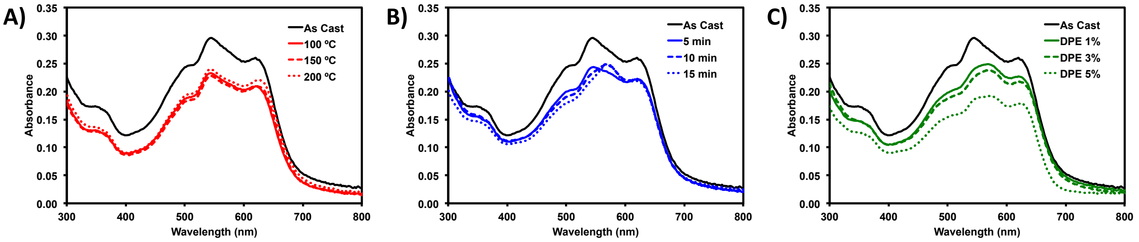

Figure 25: Optical absorption spectra of thin-films of **PBDB-T/1** blends (1:1). A) films thermal annealed, B) films solvent vapour annealed using *o*-DCB, C) films processed with DPE solvent additive. The films were spin-cast from 10 mg/mL *o*-DCB solutions at 1500 rpm for 30 s.

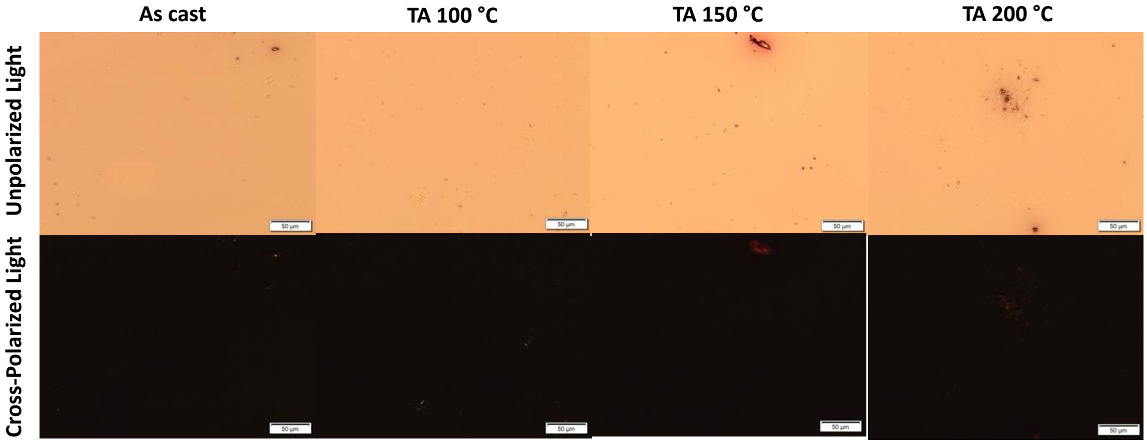

Figure 26: POM images of **PBDB-T/1** blend (1:1) thin films measured "as-cast" and after being thermally annealed. Images taken under normal and cross-polarized light. Images were taken at 20× magnification.

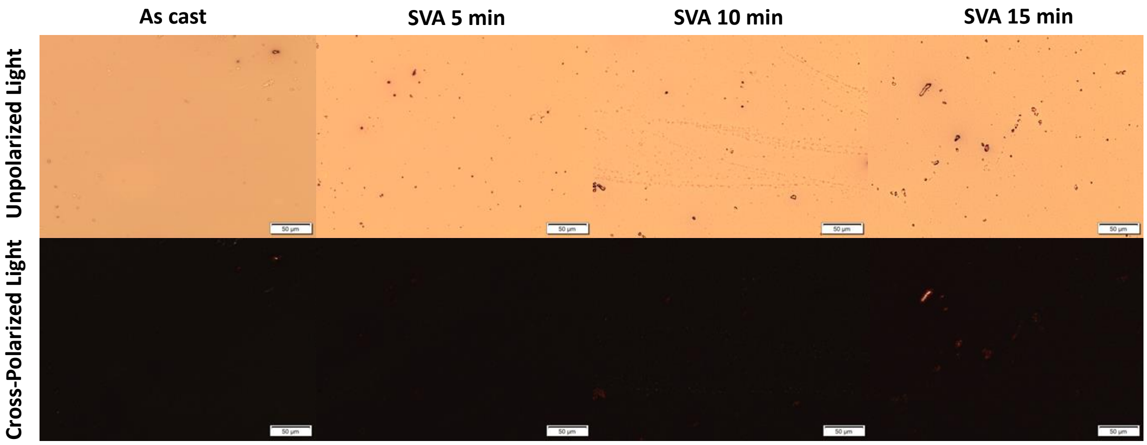

Figure 27: POM images of **PBDB-T/1** blend (1:1) thin films measured “as-cast” and after being treated with solvent vapour. Images taken under normal and cross-polarized light. Images were taken at 20× magnification.

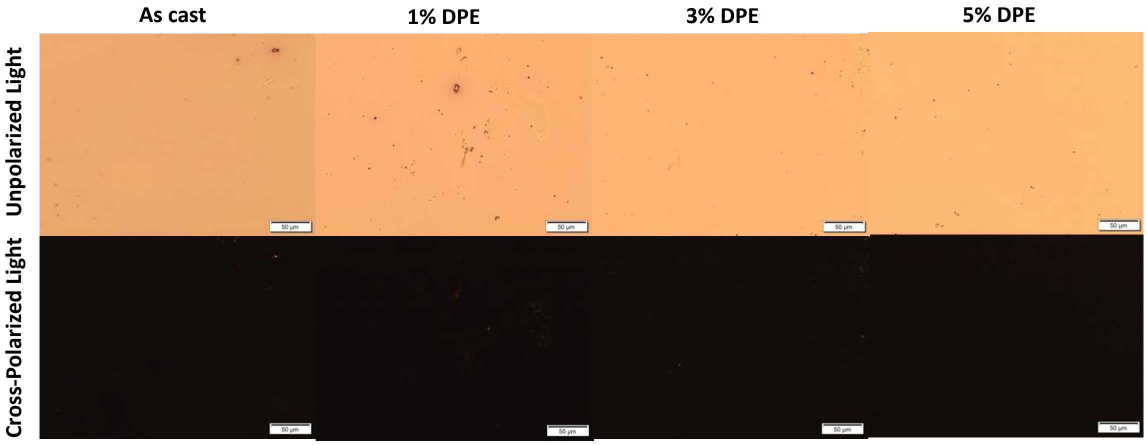

Figure 28: POM images of **PBDB-T/1** blend (1:1) thin films measured “as-cast” and processed with DPE solvent additive. Images taken under normal and cross-polarized light. Images were taken at 20× magnification.

9. Thermal Characterization

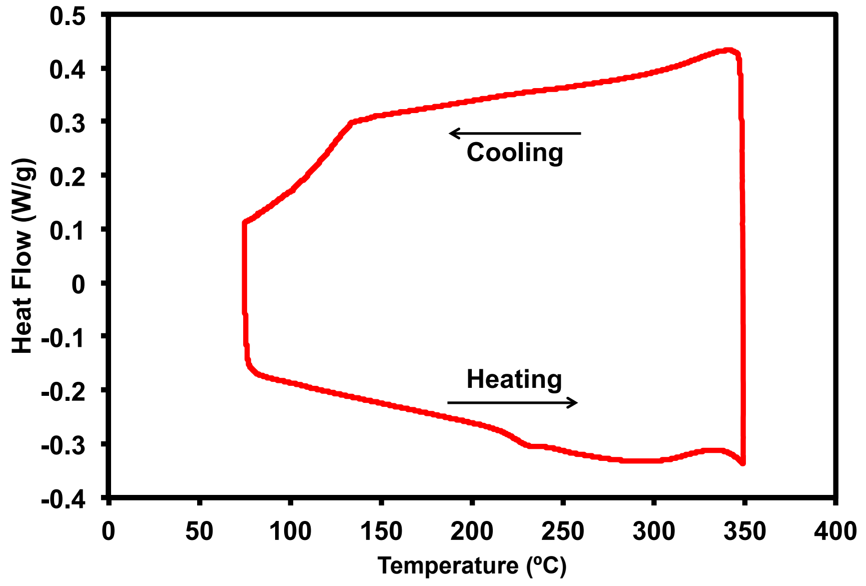

Figure S29: DSC profile for **1**.

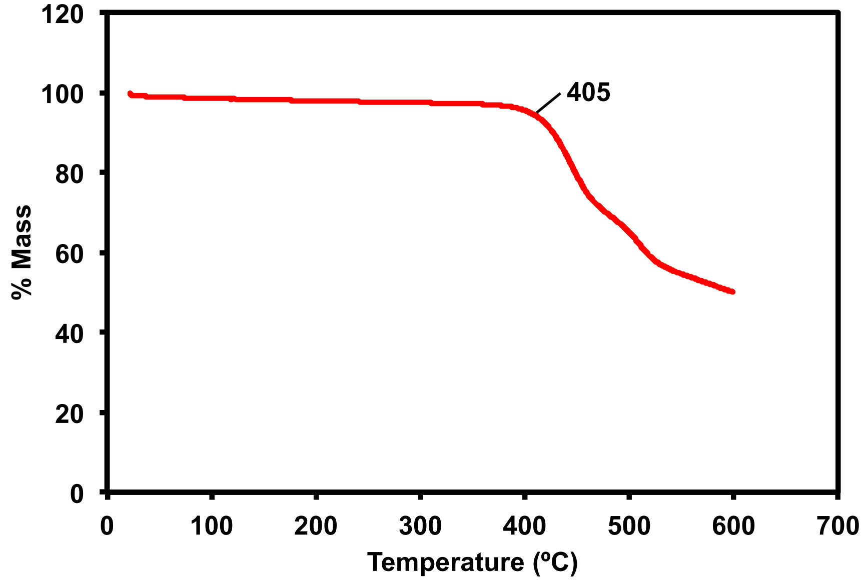

Figure S30: TGA profile for **1** with decomposition temperature shown.

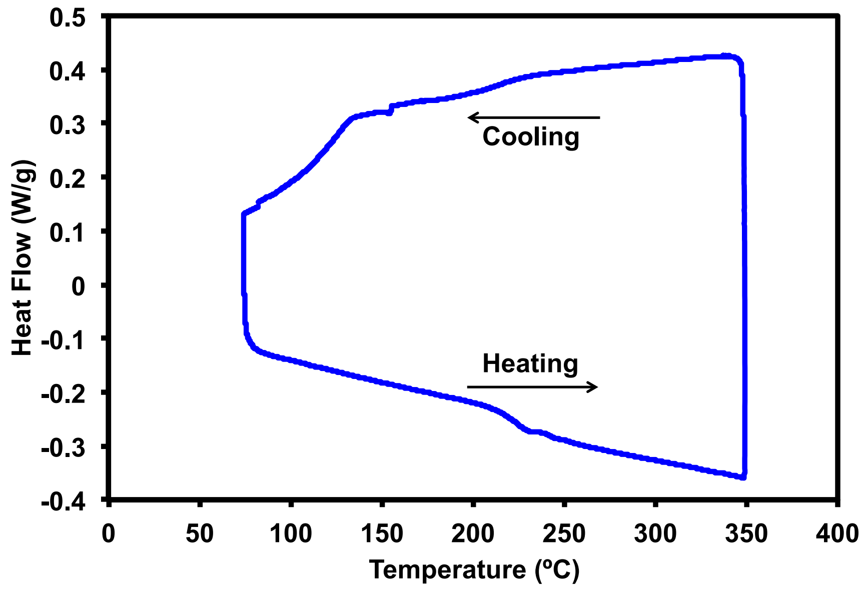

Figure S31: DSC profile for **2**.

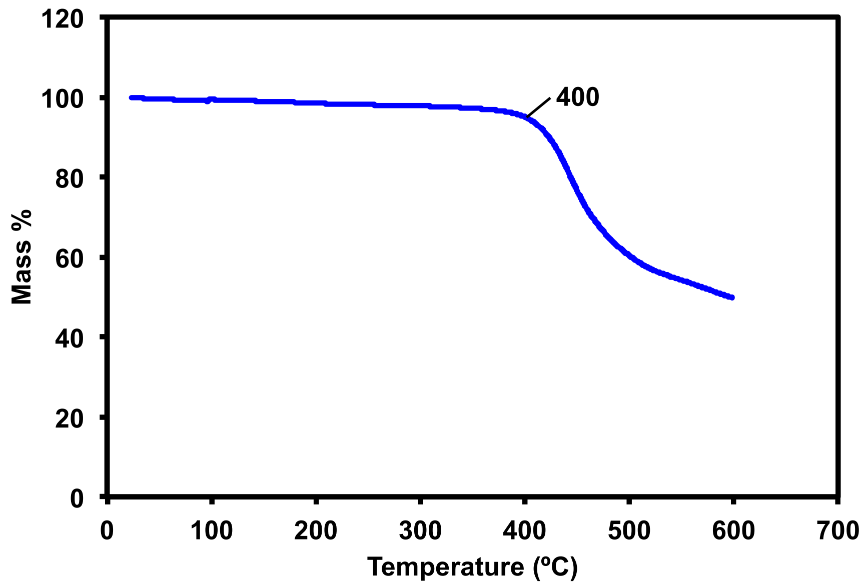

Figure S32: TGA profile for **2** with decomposition temperature shown.

10. Theoretical Modeling (reproduced from main text)

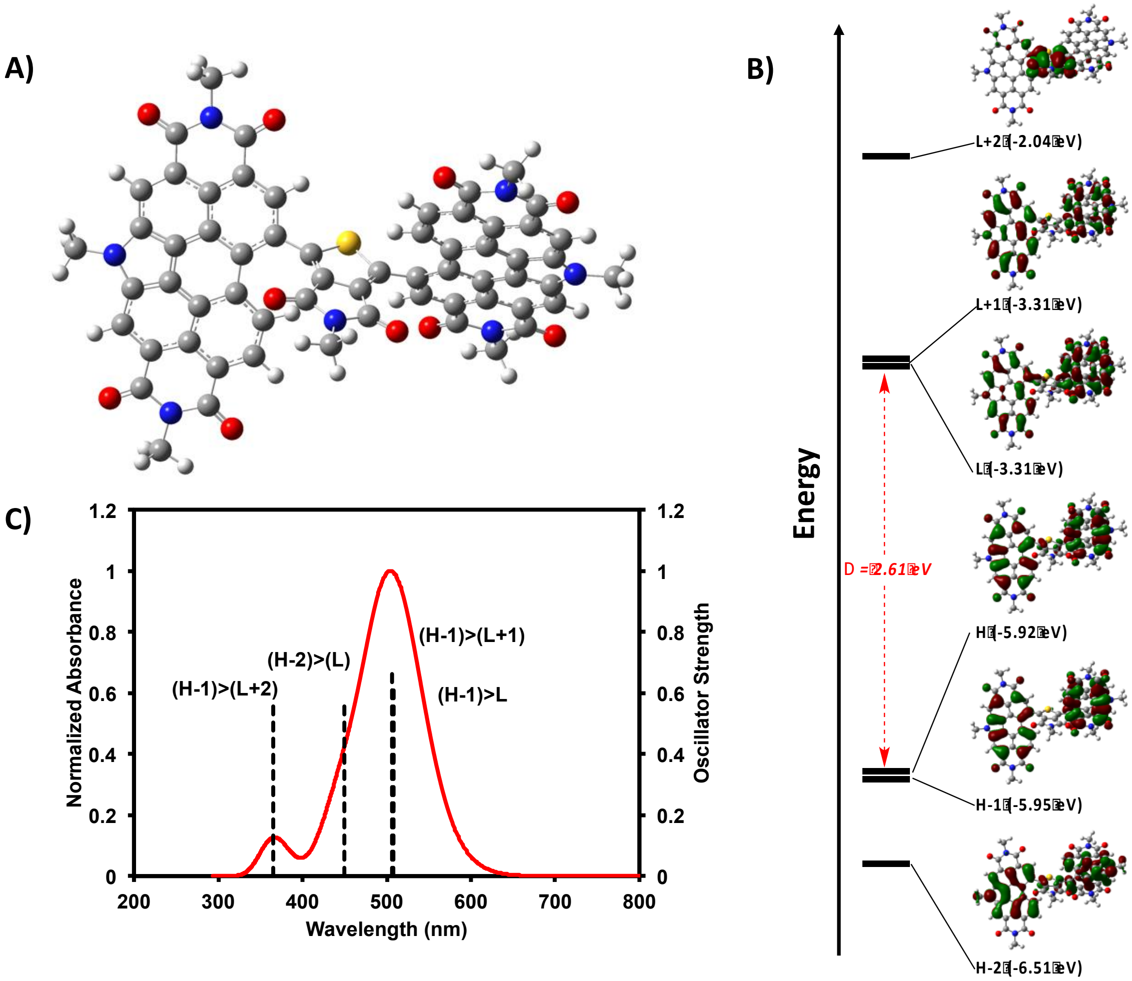

Figure S33: A) Optimized geometry for **1**. B) Calculated electronic energy levels and energy gap for **1**. C) Calculated optical absorption profile for **1**. Calculations were done on Gaussian16 [1], input files and results were visualized using GausView05 [2]. All alkyl chains were replaced with a methyl group. The B3LYP level of theory with 6-31G(d,p) [6–11] basis set were used for the calculations. TD-SCF [12] calculations were performed from the optimized geometry. The single point calculation was performed on this structure in order to generate molecular orbitals and electrostatic potential maps.

Table S4: Summary of predicted optical transitions for **$(PDI)_2TPD$**.

Compound	State	E_{opt} (eV)	λ (nm)	f	Composition
**Optimized $(PDI)_2TPD$**	S_3	2.44	508	0.130	H-1 → L (61%)<br>H → L+1 (35%)
	S_4	2.45	506	0.795	H-1 → L+1 (69%)<br>H → L (25%)<br>H-1 → L (3%)
	S_5	2.76	450	0.282	H-2 → L (56%)<br>H-3 → L+1 (37%)
	S_{15}	3.39	365	0.108	H-1 → L+2 (56%)<br>H → L+3 (23%)<br>H-10 → L (5%)<br>H-12 → L+1 (3%)<br>H-9 → L+1 (2%)

11. Organic Solar Cells

Devices were fabricated using ITO-coated glass substrates cleaned by sequentially ultra-sonicating detergent and de-ionized water, acetone, and isopropanol followed by exposure to UV/ozone for 30 minutes. ZnO was subsequently deposited as a sol-gel precursor solution in air following the method of Sun *et al.* [14]. The room temperature solution was filtered and spin-cast at a speed of 4000 rpm and then annealed at 200 °C in air for 15 min.

Active layer solutions of **PBDB-T** (Brilliant Matters, PCE12, M_w = 154 kg/mol and M_n = 76 kg/mol, batch no BM3-009-6)**,** and **1** were prepared in air with a total concentration of 10 mg/mL in *o*-dichlorobenzene (*o*-DCB) with or without a 3% (v/v) diphenyl ether (DPE) additive. Solutions were stirred overnight at room temperature and heated for 4 h at 80 °C. Active layer materials were combined in a 1:1 weight ratio and cast at room temperature in air at a speed of 1500 rpm for 60 seconds. Thermal annealing was done for 5 min at 150 °C when indicated. Solvent vapour annealing from *o*-DCB was done for 15 min.

All substrates upon casting active layers were kept in an N_2 atmosphere glovebox overnight before evaporating MoO_3 and Ag. The evaporation of 10 nm of MoO_3 followed by 100 nm of Ag were thermally deposited under vacuum ($3x10^{-6}$ Torr). The active areas of resulting devices were 0.09 cm^2. Statistics listed below for each device were tabulated from at least two substrates containing two devices each for a total of four devices.

Table S5: Organic solar cell data of 50:50 blends of **PBDB-T** and **1** cast from *o*-DCB. Best results are highlighted in bold. Averages are in italics.

**Parameters**	**V_{OC} (V)**	**J_{sc} (mA/cm^2)**	**FF (%)**	**PCE (%)**
As Cast	1.07	4.62	34.64	1.70
	1.06	4.53	34.71	1.67
	1.03	4.65	34.13	1.64
	**1.07**	**4.84**	**35.15**	**1.81**
	1.06	*4.66*	*34.66*	*1.71*
TA 150 °C 5 min	1.06	4.96	35.76	1.89
	1.08	4.88	36.90	1.94
	**1.07**	**5.04**	**38.59**	**2.09**
	1.08	4.78	37.29	1.92
	1.07	*4.91*	*37.14*	*1.96*
SVA *o*-DCB 15 min	1.03	3.81	36.57	1.43
	**1.03**	**4.00**	**36.52**	**1.50**
	1.03	3.85	36.13	1.43
	1.02	4.01	36.09	1.48
	1.03	*3.92*	*36.33*	*1.46*
DPE 3%	1.04	6.91	43.30	3.12
	1.05	6.91	42.82	3.10
	**1.05**	**7.40**	**42.37**	**3.28**
	1.04	6.85	42.91	3.07
	1.04	*7.02*	*42.85*	*3.14*

12. References

1. Frisch, M.; Trucks, G.; Schlegel, H.; Scuseria, G.; Robb, M.; Cheeseman, J.; Scalmani, G.; Barone, V.; Mennucci, B.; Petersson, G.; Nakatsuji, H.; Caricato, M.; Li, X.; Hratchian, H.; Izmaylov, A.; Bloino, J.; Zheng, G.; Sonnenberg, J.; Hada, M.; Ehara, M.; Toyota, K.; Fukuda, R.; Hasegawa, J.; Ishida, M.; Nakajima, T.; Honda, Y.; Kitao, O.; Nakai, H.; Vreven, T.; Montgomery, J.; Peralta, J.; Ogliaro, F.; Bearpark, M.; Heyd, J.; Brothers, E.; Kudin, K.; Staroverov, V.; Kobayashi, R.; Normand, J.; Raghavachari, K.; Rendell, A.; Burant, J.; Iyengar, S.; Tomasi, J.; Cossi, M.; Rega, N.; Millam, J.; Klene, M.; Knox, J.; Cross, J.; Bakken, V.; Adamo, C.; Jaramillo, J.; Gomperts, R.; Stratmann, R.; Yazyev, O.; Austin, A.; Cammi, R.; Pomelli, C.; Ochterski, J.; Martin, R.; Morokuma, K.; Zakrzewski, V.; Voth, G.; Salvador, P.; Dannenberg, J.; Dapprich, S.; Daniels, A.; Farkas; Foresman, J.; Ortiz, J.; Cioslowski, J.; Fox, D. Gaussian 16, Revision A.03. *Gaussian 16 Revis. A03 Gaussian Inc Wallingford CT* **2016**.
2. *GaussView Version 5*;
3. Becke, A. D. Density-functional exchange-energy approximation with correct asymptotic behavior. *Phys. Rev. A* **1988**, *38*, 3098–3100.
4. Lee, C.; Yang, W.; Parr, R. G. Development of the Colle-Salvetti correlation-energy formula into a functional of the electron density. *Phys. Rev. B* **1988**, *37*, 785–789.
5. Miehlich, B.; Savin, A.; Stoll, H.; Preuss, H. Results obtained with the correlation energy density functionals of becke and Lee, Yang and Parr. *Chem. Phys. Lett.* **1989**, *157*, 200–206, doi:10.1016/0009-2614(89)87234-3.
6. Hehre, W. J.; Ditchfield, R.; Pople, J. A. Self—Consistent Molecular Orbital Methods. XII. Further Extensions of Gaussian—Type Basis Sets for Use in Molecular Orbital Studies of Organic Molecules. *J. Chem. Phys.* **1972**, *56*, 2257–2261, doi:http://dx.doi.org/10.1063/1.1677527.
7. Hariharan, P. C.; Pople, J. A. The influence of polarization functions on molecular orbital hydrogenation energies. *Theor. Chim. Acta* **1973**, *28*, 213–222, doi:10.1007/BF00533485.
8. Francl, M. M.; Pietro, W. J.; Hehre, W. J.; Binkley, J. S.; Gordon, M. S.; DeFrees, D. J.; Pople, J. A. Self-consistent molecular orbital methods. XXIII. A polarization-type basis set for second-row elements. *J. Chem. Phys.* **1982**, *77*, 3654–3665, doi:http://dx.doi.org/10.1063/1.444267.
9. Binning, R. C.; Curtiss, L. A. Compact contracted basis sets for third-row atoms: Ga–Kr. *J. Comput. Chem.* **1990**, *11*, 1206–1216, doi:10.1002/jcc.540111013.
10. Rassolov, V. A.; Pople, J. A.; Ratner, M. A.; Windus, T. L. 6-31G* basis set for atoms K through Zn. *J. Chem. Phys.* **1998**, *109*, 1223–1229, doi:http://dx.doi.org/10.1063/1.476673.
11. Rassolov, V. A.; Ratner, M. A.; Pople, J. A.; Redfern, P. C.; Curtiss, L. A. 6-31G* basis set for third-row atoms. *J. Comput. Chem.* **2001**, *22*, 976–984, doi:10.1002/jcc.1058.
12. Bauernschmitt, R.; Ahlrichs, R. Treatment of electronic excitations within the adiabatic approximation of time dependent density functional theory. *Chem. Phys. Lett.* **1996**, *256*, 454–464, doi:10.1016/0009-2614(96)00440-X.

13. Pommerehne, J.; Vestweber, H.; Guss, W.; Mahrt, R. F.; Bässler, H.; Porsch, M.; Daub, J. Efficient two layer leds on a polymer blend basis. *Adv. Mater.* **1995**, *7*, 551–554, doi:10.1002/adma.19950070608.
14. Sun, Y.; Seo, J. H.; Takacs, C. J.; Seifter, J.; Heeger, A. J. Inverted Polymer Solar Cells Integrated with a Low-Temperature-Annealed Sol-Gel-Derived ZnO Film as an Electron Transport Layer. *Adv. Mater.* **2011**, *23*, 1679–1683, doi:10.1002/adma.201004301.
